# Ion cocktail therapy for myocardial infarction by synergistic regulation of both structural and electrical remodeling

**DOI:** 10.1002/EXP.20230067

**Published:** 2023-11-23

**Authors:** Yumei Que, Jiaxin Shi, Zhaowenbin Zhang, Lu Sun, Hairu Li, Xionghai Qin, Zhen Zeng, Xiao Yang, Yanxin Chen, Chong Liu, Chang Liu, Shijie Sun, Qishu Jin, Yanxin Zhang, Xin Li, Ming Lei, Chen Yang, Hai Tian, Jiawei Tian, Jiang Chang

**Affiliations:** ^1^ Joint Centre of Translational Medicine The First Affiliated Hospital of Wenzhou Medical University Wenzhou Zhejiang China; ^2^ Zhejiang Engineering Research Center for Tissue Repair Materials Wenzhou Institute University of CAS Wenzhou China; ^3^ Department of Ultrasound The Second Affiliated Hospital of Harbin Medical University Harbin China; ^4^ Shanghai Institute of Ceramics Chinese Academy of Sciences (CAS) Shanghai China; ^5^ Center of Materials Science and Optoelectronics Engineering University of CAS Beijing China; ^6^ Department of Cardiovascular Surgery Peking University Shenzhen Hospital Shenzhen China; ^7^ Future Medical Laboratory The Second Affiliated Hospital of Harbin Medical University Harbin China; ^8^ Department of Cardiovascular Surgery The Second Affiliated Hospital of Harbin Medical University Harbin China; ^9^ Department of Pharmacology University of Oxford Oxford UK

**Keywords:** ion cocktail, myocardial infarction, remodeling

## Abstract

Myocardial infarction (MI) is a leading cause of death worldwide. Few drugs hold the ability to depress cardiac electrical and structural remodeling simultaneously after MI, which is crucial for the treatment of MI. The aim of this study is to investigate an effective therapy to improve both electrical and structural remodeling of the heart caused by MI. Here, an “ion cocktail therapy” is proposed to simultaneously reverse cardiac structural and electrical remodeling post‐MI in rats and minipigs by applying a unique combination of silicate, strontium (Sr) and copper (Cu) ions due to their specific regulatory effects on the behavior of the key cells involved in MI including angiogenesis of endothelial cells, M2 polarization of macrophages and apoptosis of cardiomyocyte. The results demonstrate that ion cocktail treatment attenuates structural remodeling post‐MI by ameliorating infarct size, promoting angiogenesis in both peri‐infarct and infarct areas. Meantime, to some extent, ion cocktail treatment reverses the deteriorative electrical remodeling by reducing the incidence rate of early/delayed afterdepolarizations and minimizing the heterogeneity of cardiac electrophysiology. This ion cocktail therapy reveals a new strategy to effectively treat MI with great clinical translation potential due to the high effectiveness and safety of the ion cocktail combination.

## Graphical abstract

The ion cocktail, consisting of silicate, strontium and copper could attenuate both electrical and structural remodeling after myocardial infarction in rat and Bama minipigs. The therapeutic effect of ion cocktail on myocardial infarction may account for that different ions in the cocktail have different functions to regulate cellular behaviors.

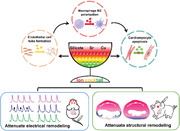

## 1 INTRODUCTION

Myocardial infarction (MI) is leading causes of lethality world‐wide, while the development of therapeutic drugs in this area remains a daunting challenge. Although pharmacological therapy (e.g., aspirin or lidocaine) can improve blood flow or relieve arrhythmias after MI, these treatments are generally beneficial either only for electrical or structural remodeling and show limited benefit to patients.^[^ ^1^ ^]^ Biotherapies such as stem cell and gene therapy have been developed to repair infarcted heart by improving angiogenesis and cardiomyocyte survival, as well as regulating inflammatory responses and cardiac electrical conduction, but their effect on electrical remodeling might not be positive or even increase the risk of arrhythmias.^[^ ^2^ ^]^

**SCHEME 1:**
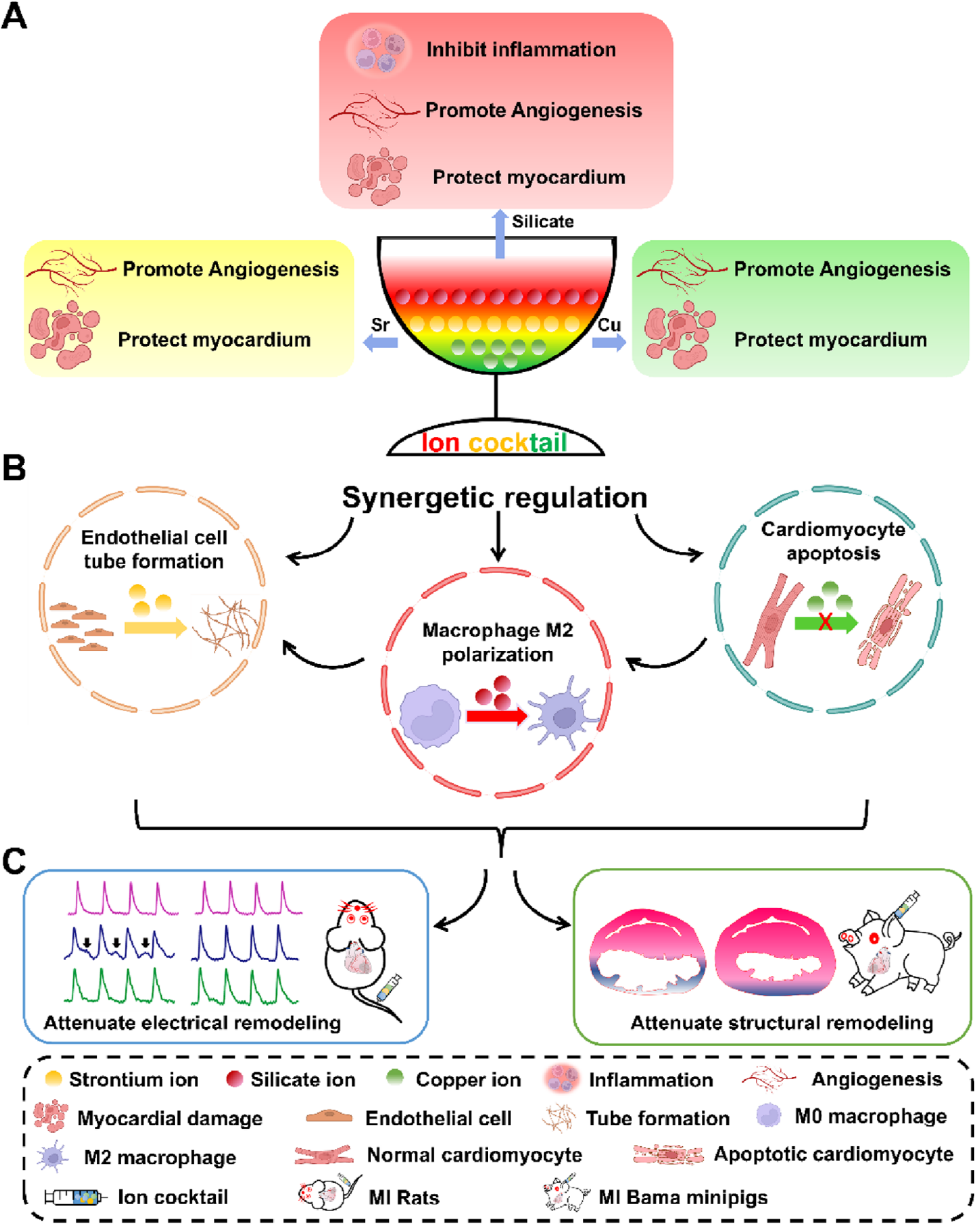
Schematic diagram of ion cocktail therapy for MI. (A) Different ions in the cocktail have different functions to regulate pathological process of MI, including ischemic injury, acute inflammation and myocardial damage. Silicate ions could inhibit acute inflammation, promote angiogenesis and protect myocardium while Sr and Cu ions could promote angiogenesis and protect myocardium simultaneously. (B) Ion cocktail could synergistically regulate the cellular behaviors during MI, including promoting endothelial cell tube formation and macrophage M2 polarization, as well as inhibiting cardiomyocyte apoptosis. (C) Ion cocktail could attenuate both electrical and structural remodeling after MI. MI: myocardial infarction. This scheme was created with BioRender.com.

Our previous work has shown that silicate ceramics‐based biomaterials could effectively repair the myocardium after MI and improve cardiac function by facilitating intercellular communication and enhancing the expression of gap junction protein 43 (Cx43), as well as inhibiting cardiomyocyte apoptosis, and promoting Ca^2+^ transients.^[^ ^3^ ^]^ Strontium (Sr) and Copper (Cu) ions showed a unique effect on enhancing angiogenesis and inhibiting cardiomyocyte apoptosis.^[^ ^4^ ^]^ Besides, Sr ions could also regulate M2 polarization of macrophages.^[^ ^5^ ^]^ However, the single ion treatment may not be strong enough in MI treatment. Although Sr and Cu have been reported to prolong the action potential^[^ ^6^ ^]^ and block Ca^2+^‐permeable cationic channel (TRPM2),^[^ ^7^ ^]^ respectively, it is unknown about the effect of these ions, in particular the combination of these ions on cardiac electrical remodeling post‐MI. Here, in this study, we for the first time proposed an “ion cocktail therapy” consisting of silicate, Sr and Cu ions and hypothesized that the enhanced therapeutic effect may be obtained by the specific regulatory effect of different ions and in particular the synergetic regulatory effects of different ion combinations, which eventually attenuates both structural and electrical remodeling of the injured heart. The effectiveness of the “ion cocktail therapy” was proven by using both in vivo rat and Bama minipig MI models with an attention on attenuating both cardiac electrical and structural remodeling after MI. The possible mechanisms of the ion cocktail were explored by in vitro experiment to evaluate the synergistic regulatory effects of different ion combinations and compared with the activity of each single ions on different cells (endothelial cells, macrophages and cardiomyocytes) involved in the key steps of myocardial repair including pro‐angiogenesis, anti‐inflammation and anti‐apoptosis (Scheme 1).

## 2 RESULTS

### 2.1 The ion cocktail reduces the incidence rate of early and delayed afterdepolarizations (EAD/DAD) and minimizes the heterogeneity of cardiac electrophysiology post‐MI

**FIGURE 1:**
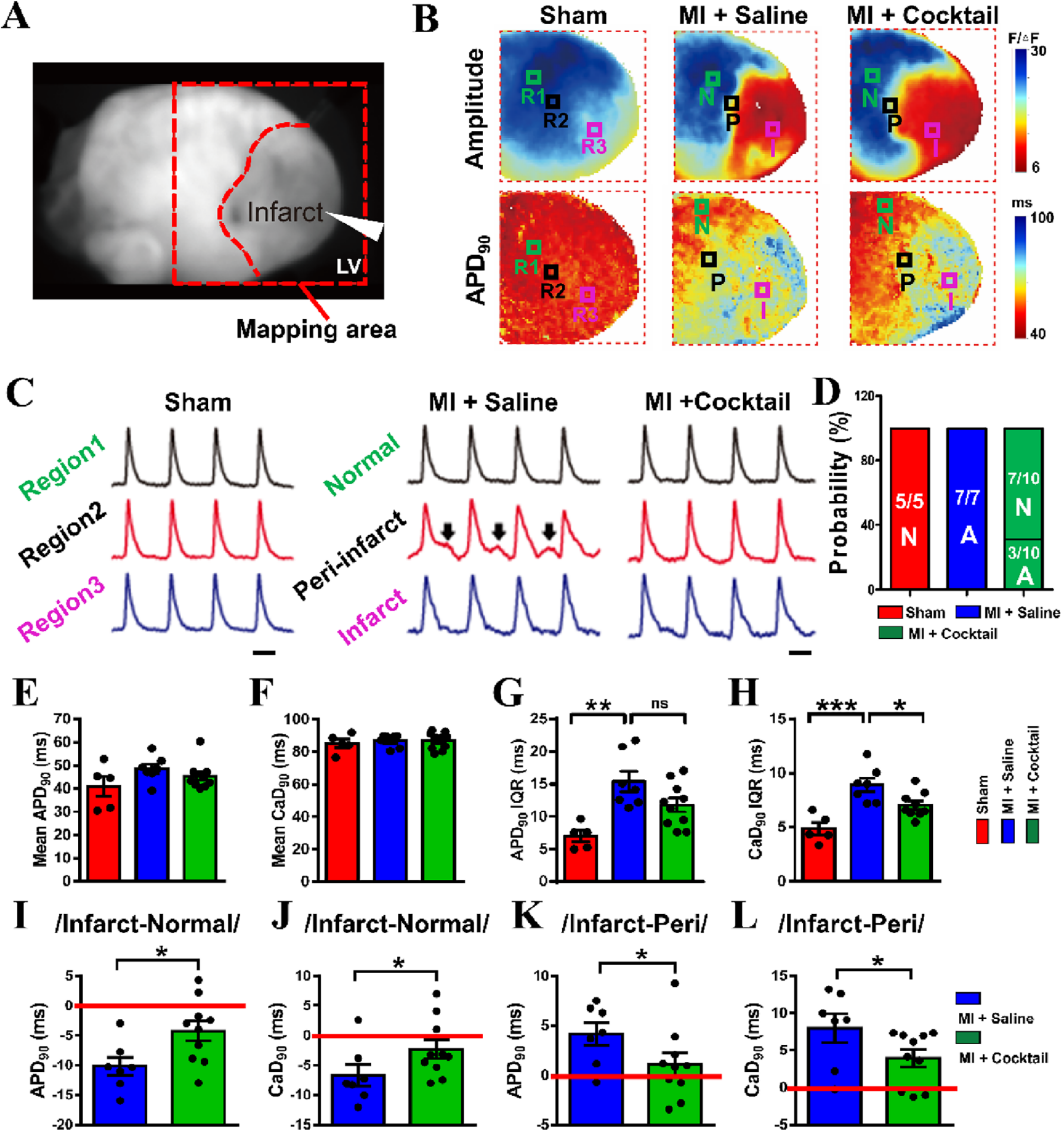
The ion cocktail reduces the incidence rate of EAD/DAD and the heterogeneity of cardiac electrophysiology in rats 28 days post‐MI. (A) The representative image of a heart is prepared for optical mapping. The red dotted box indicates the ventricle; infarct region is in the apical left ventricle; the stimulating electrode is at the apex of the heart (the white triangle). (B) The representative maps of amplitude (top) and APD90 (bottom) in the ventricle of the heart among different groups. Region 1 (R1) or normal area (N) is indicated with green square, region 2 (R2) or peri‐infarct area (P) is indicated with black square, and region 3 (R3) or infarct area (I) is indicated with purple square. The heatmap scale bar indicates the amplitude (*F*/∆*F*, top) or action potential duration (ms, top). (C) Representative traces of optical action potential (AP) from different areas among different groups. Black arrow indicates EAD/DAD. Scale bar, 100 ms. (D) The incidence rate of EAD/DAD. N represents no EAD/DAD appeared; A represents EAD/DAD appeared. The quantified (E) APD90, (F) CaD90, (G) IQR of APD90 (a measure of APD90 dispersion). (H) IQR of CaD90 (a measure of CaD90 dispersion) calculated from optical maps among different groups. The difference value of (I) APD90 and (J) CaD90 between infarct area and normal area. (K) APD90 and (L) CaD90 between infarct area and peri‐infarct area (*n* = 5 for Sham, *n* = 7 for MI + Saline and *n* = 10 for MI + Cocktail). *p < 0.05, **p < 0.01 or ***p < 0.001. APD90: action potential duration of 90%. CaD90: Ca^2+^ transient duration of 90%. EAD: early after depolarization. DAD: delay after depolarization.

To explore whether ion cocktail, which consists of silicate, Sr and Cu, could influence cardiac electrophysiology, we first investigated the effects of the ion cocktail on cardiac electrophysiology by conducting optical mapping in MI (28 days of ligation) rat model by intravenous injection of ion cocktail. The ion cocktail for in vivo experiments is prepared in saline with the final concentration of 105 µg mL^−1^ for silicate, 250 µg mL^−1^ for Sr and 20 µg mL^−1^ for Cu. No precipitation of ion cocktail is formed as shown in Figure S1. The body weight was first calculated (Figure S2), which showed that the ion cocktail had barely effects on body weight as compared to control group. Then, the optical mapping analysis was conducted. As shown Figure 1A, the pacing electrode was positioned on the apex of left ventricle (LV) epicardium, and the entire heart ventricle area was mapped for further analysis. Figure 1B (top) shows representative maps of amplitude and APD90 (top, action potential duration of 90%) in different groups, in which the normal area (N), peri‐infarct area (P) and infarct area (I) of MI heart were highlighted with green, black and purple, respectively. By analyzing the traces of optical AP in different areas (N, P and I) of each heart (Figure 1C), we found that, in contrast to MI group, the incidence rate of EAD/DAD, a precursor of arrhythmogenesis,^[^ ^8^ ^]^ showed a sharp increase from 0% (0 out of 5) to 100% (7 out of 7) in peri‐infarct area, while the treatment of ion cocktail reduced the EAD/DAD incidence rate to 30% (3 out of 10) (Figure 1D).

Since the dispersion of APD90 or CaD90 directly reflects the severity of MI, we carefully analyzed the APD90/CaD90 dispersion through the measurement of interquartile range (IQR), which is one of the dispersion parameters based on lower and upper quartiles across the entire ventricle.^[^ ^9^ ^]^ As shown in Figure 1E–H, although there were no significant differences in the mean APD90 or CaD90 among different groups, the IQR of both APD90 and CaD90 significantly increased in MI + Saline group (APD90 IQR: 15.41 ± 1.58 ms and CaD90 IQR: 8.91 ± 0.61 ms) as compared to Sham group (APD90 IQR: 7.0 ± 0.87 ms and CaD90 IQR: 4.86 ± 0.58 ms), indicating the success of MI model. More interestingly, after treatment of ion cocktail, the IQR of APD90 and CaD90 significantly decreased to 11.77 ± 1.07 ms and 7.03 ± 0.39 ms, respectively, demonstrating a reduced heterogeneity of cardiac electrophysiology. Interestingly, in addition to dispersion of APD90 or CaD90, we noticed that the APD90 or CaD90 values for different areas of the heart (infarct and normal area or infarct and peri‐infarct area) in MI group are different as compared to Sham group (Table S1, Supporting Information), indicating a decreased uniformity of the cardiac electrophysiology. Therefore, we introduced an additional parameter, the difference value (DV) of APD90 or CaD90 between infarct area and peri‐infarct or non‐infarct area (Normal area) of heart to express the severity of cardiac electrophysiology uniformity. It is clear to see that the DV of APD90 or CaD90 in MI group was significantly increased as compared to Sham group (Table S2, Supporting Information). In contrast, after ion cocktail treatment, the DV of APD90 or CaD90 was decreased as compared to MI + Saline group, indicating a successful return of cardiac electrophysiology uniformity (Figure 1I–L). Similar results were also observed when an isoproterenol (ISO) stimulation was applied, as either IQR of APD90/CaD90 across the entire ventricle or DV of APD90/CaD90 between different areas declined after the treatment of ion cocktail (Figures S3 and S4), indicating the stability of the ion cocktail in regulating the dispersion of APD90 or CaD90.

### 2.2 The ion cocktail reduces the incidence rate of ventricular fibrillation in rats post‐MI

**FIGURE 2:**
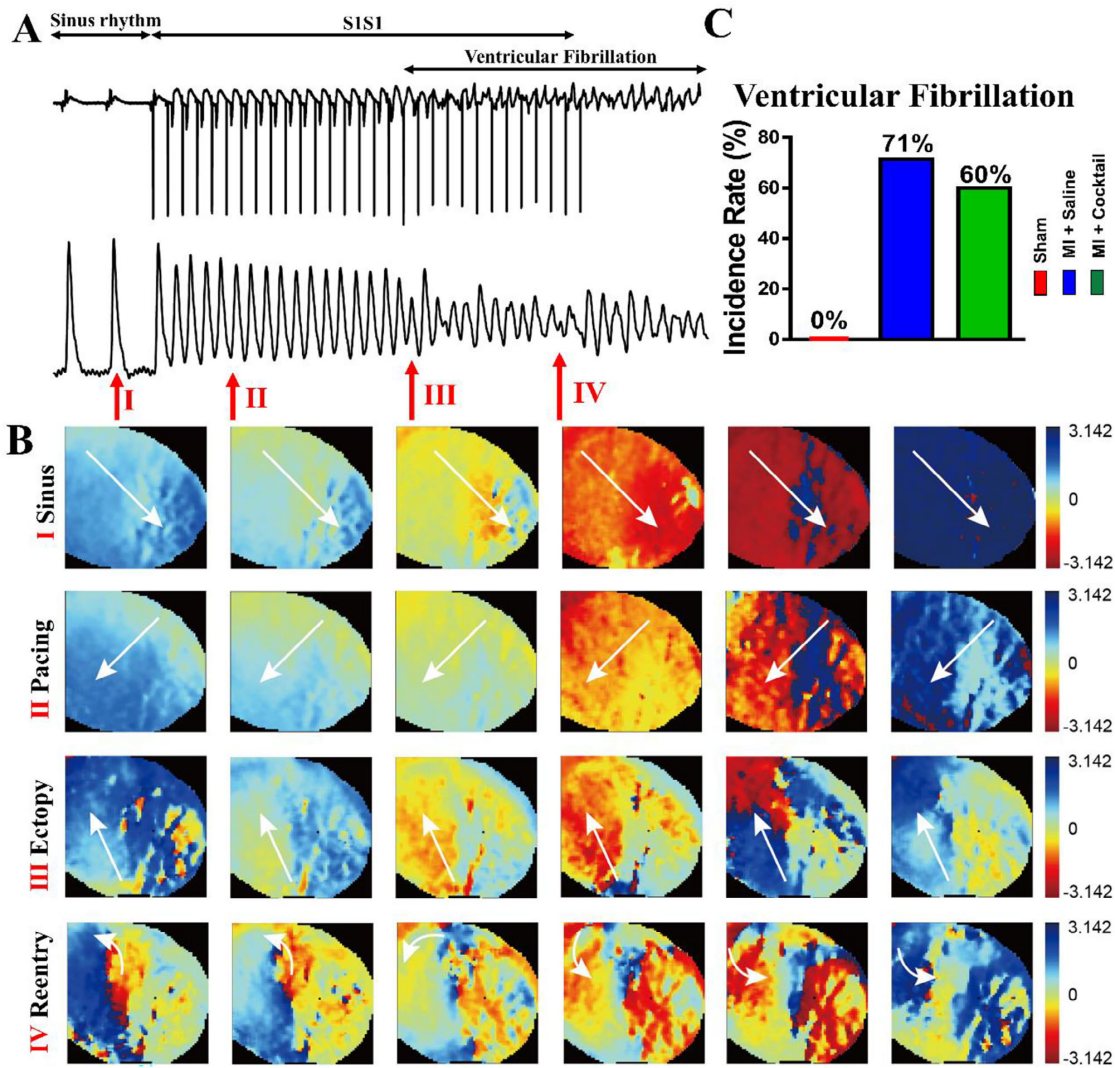
The ion cocktail reduces the incidence rate of ventricular fibrillation in rats 28 days post‐MI. (A) Representative electrocardiograph and AP trace recorded, showing sinus rhythm, S1S1 pacing, ectopy and ventricular fibrillation in MI rat. The number I, II, III and IV with the red arrow represent the location of sinus, pacing, ectopy and reentry, respectively. (B) The phase maps in MI rat, showing sinus, pacing, ectopy and reentry. The arrows represent the direction of electrical conduction. (C) Incidence rate of ventricular fibrillation in different groups (*n* = 5 for Sham, *n* = 7 for MI + Saline and *n* = 10 for MI + Cocktail).

The frequency of ventricular fibrillation (VF), a form of severe arrhythmia,^[^ ^10^ ^]^ was measured using programmed stimulation with an S1S1 interval (30 times, S1 stimuli of 150 ms). The electrocardiogram (ECG) and AP trace records showed sinus rhythm, S1S1 pacing, ectopy and VF in MI rat, indicating that the severe ventricular fibrillation occurred after 28 days post‐MI (Figure 2A). Similarly, the phase maps showed that the reentry is obvious in the heart of MI while the sinus, pacing and ectopy were normal (Figure 2B). Furthermore, the quantitative incidence rate of VF was increased from 0% in Sham group to 71% in MI + Saline group, while the ion cocktail injection reduced VF to 60% in MI + Cocktail group (Figure 2C), confirming that ion cocktail has anti‐arrhythmia effect.

### 2.3 The ion cocktail ameliorates myocardial fibrosis and promotes angiogenesis in rats post‐MI

**FIGURE 3:**
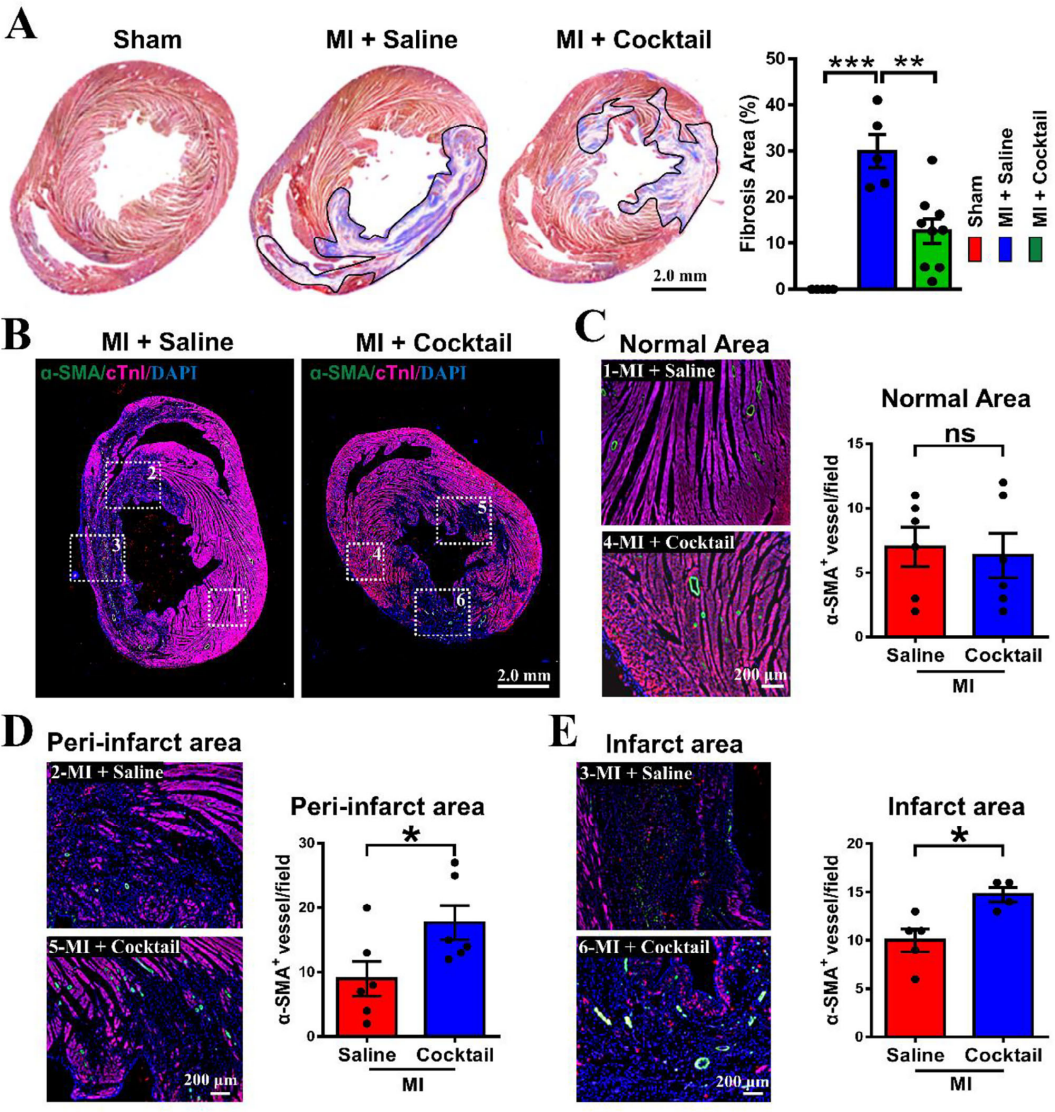
The ion cocktail ameliorates cardiac fibrosis and promotes angiogenesis in rats 28 days post‐MI. (A) Representative Masson's trichrome staining images and quantification of fibrosis area in different groups. The infarct area was highlighted with black line (*n* = 4 for Sham, *n* = 5 for MI + Saline and *n* = 9 for MI + Cocktail). (B) Representative immunofluorescence staining images of α‐SMA (green), cTnI (rosy) and DAPI (blue). The white dotted box represents different regions of the heart. 1 and 4 represent normal area, 2 and 5 represent peri‐infarct area, 3 and 6 represent infarct area. (C) Representative immunofluorescence staining images (magnified from the white dotted box 1 and 4 in (B)) and the quantification of α‐SMA^+^ vessels in normal area (*n* = 6). (D) Representative immunofluorescence staining images (magnified from the white dotted boxes 2 and 5 in (B) and the quantification of α‐SMA^+^ vessels in peri‐infarct area (*n* = 6). (E) Representative immunofluorescence staining images (magnified from the white dotted boxes 3 and 6 in (B) and the quantification of α‐SMA^+^ vessels in infarct area (*n* = 5 for MI + Saline and *n* = 4 for MI + Cocktail). *p < 0.05, **p < 0.01 or ***p < 0.001. ns: no significance. α‐SMA: Alpha‐smooth muscle actin. cTnI: cardiac troponin I.

The histological analyses were further implemented to verify the protective effect of ion cocktail in MI rats, and Masson's trichrome staining was performed to determine the ratio of scar area to ventricle. As shown in Figure 3A, abundant fibrotic myocardial tissues appeared in MI + Saline group, and the ion cocktail treatment reduced fibrosis in MI + Cocktail group. Such a trend was confirmed by quantitative analysis, which showed that the fibrosis area in MI + Saline group was 29.99 ± 3.65%, and the ion cocktail treatment significantly decreased the infarct area ratio to 12.62 ± 2.71% (MI + Cocktail group). Moreover, the pro‐angiogenetic effect of ion cocktail was measured using immunofluorescence staining of alpha‐smooth muscle actin (α‐SMA, a vascular smooth muscle cell‐specific marker), and cardiac troponins T (cTnI, a cardiac‐specific protein) (Figure 3B). The result revealed that the number of α‐SMA^+^ vessels was comparable in normal areas between MI + Saline (7 ± 2) and MI + Cocktail (6 ± 2) groups (Figure 3C). However, in both peri‐infarct and infarct areas, the number of α‐SMA^+^ vessels in MI + Cocktail group (peri‐infarct: 18 ± 3; infarct: 15 ± 1) was significantly higher than that in MI + Saline group (peri‐infarct: 9 ± 3; infarct: 10 ± 1) (Figure 3D,E). These results demonstrated that ion cocktail significantly enhanced vascularization in both peri‐infarct and infarct areas of MI rats.

### 2.4 The ion cocktail alleviates MI and improves cardiac function in Bama minipigs

**FIGURE 4:**
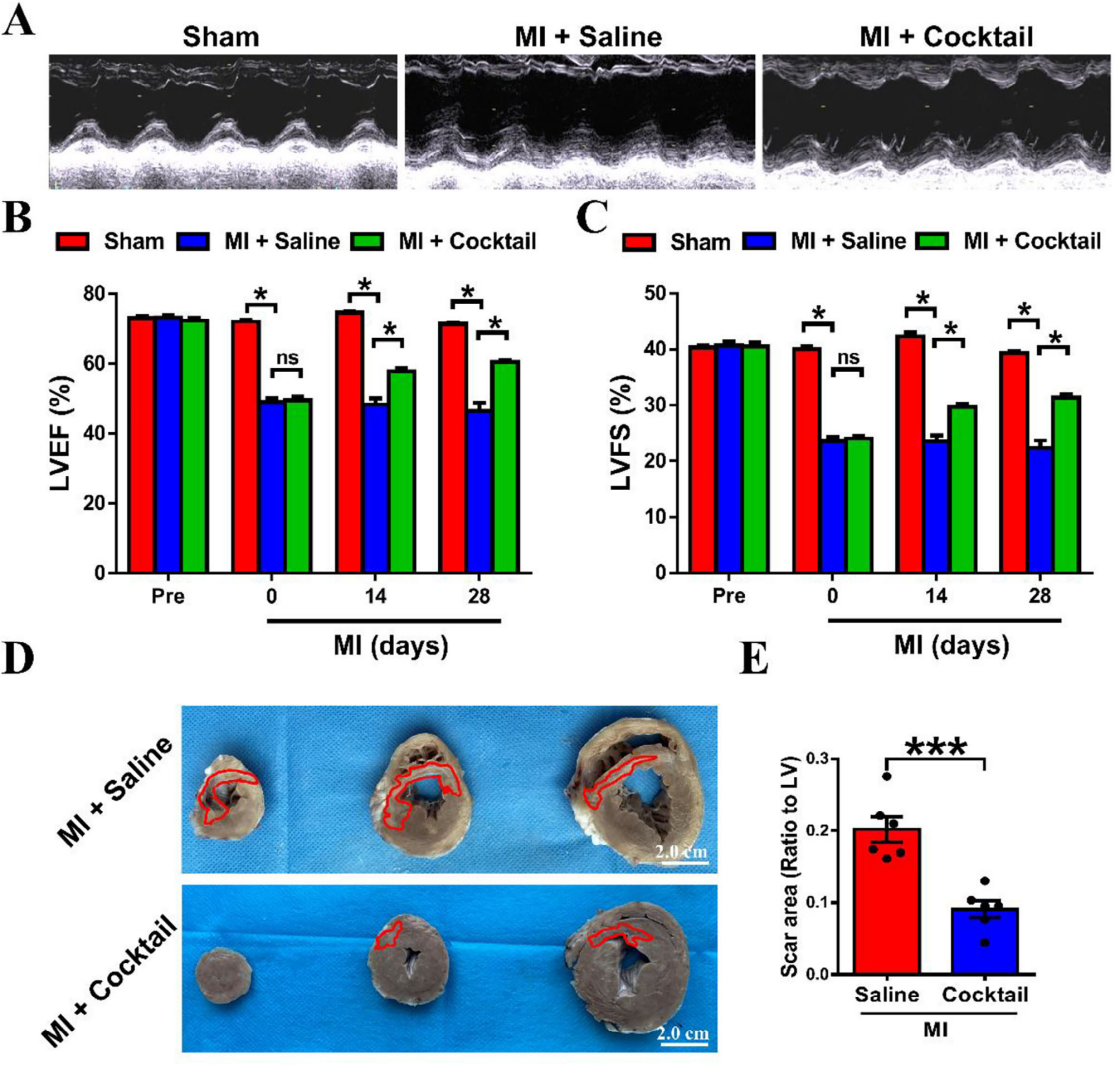
The ion cocktail improves cardiac function and decreases scar area in Bama miniature pigs 28 days post‐MI. (A) Representative echocardiographic images obtained from LV of different groups. The percentages of (B) LVEF, and (C) LVFS measured from echocardiography in different groups at pre‐MI, and different days (0, 14 and 28) post‐MI (*n* = 3 for Sham, *n* = 6 for MI + Saline and MI + Cocktail). (D) Representative cross‐section images of the heart in MI + Saline and MI + Cocktail groups. The sections in each panel are obtained from one heart and arranged from the apex cordis to base of the LV at 28 days post‐MI. The scar regions in the sections are highlighted by red lines. (E) The quantification of scar areas (presented as the ratio of scar regions vs LV) in MI + Saline and MI + Cocktail groups (*n* = 6). *p < 0.05 or ***p < 0.001. ns: no significance. LVEF: left ventricle ejection fraction. LVFS: left ventricle fractional shortening.

Although rat studies are valuable, they have limitations for translational studies because of the distinctly different cardiovascular anatomy and physiology between rodents and humans.^[^ ^11^ ^]^ Therefore, we further performed studies to evaluate cardiac beneficial effect of ion cocktail in a Bama minipig model of MI. Representative M‐model echocardiography images and the corresponding quantitative analysis of the LV function including left ventricular ejection fraction (LVEF) and left ventricular fractional shorting (LVFS) at day 28 after MI for each group are shown in Figure 4A–C. The results demonstrated that MI caused a substantial reduction in LV function (immediately after MI, 0 day), as indicated by a dramatic decline in both LVEF (MI + Saline: from 73.67% to 49.00%; MI + Cocktail: from 72.33% to 49.33%) and LVFS (MI + Saline: from 40.67% to 23.67%; MI + Cocktail: from 40.33% to 24.00%). After the treatment of ion cocktail for 14 days, both LVEF (from 49.33% to 57%) and LVFS (from 24% to 29.45%) were recovered in MI + Cocktail group, whereas no significant changes happened in MI + Saline group. More interestingly, the recovered LV function (LVEF and LVFS) in MI + Cocktail group did not decrease, but even further slightly increased 14 days after stopping ion cocktail injection (28 days post‐MI), indicating the sustained effectiveness of ion cocktail. Taken together, these results suggested that the ion cocktail was able to improve the contraction function and beating compliance of infarcted LVs. Beyond that, the gross inspection of hearts showed that scars were present in the sections at the anteroseptal and anterior LV walls in both MI + Saline and MI + Cocktail groups, but the scar in MI + Cocktail groups smaller (Figure 4D). Quantitative morphological analysis of heart sections revealed that the percentage of scar area to ventricle was significantly decreased from 20.18 ± 1.77% in MI + Saline to 9.09 ± 1.17% in MI + Cocktail group (Figure 4E), indicating the cardiac repair function of the ion cocktail.

**FIGURE 5:**
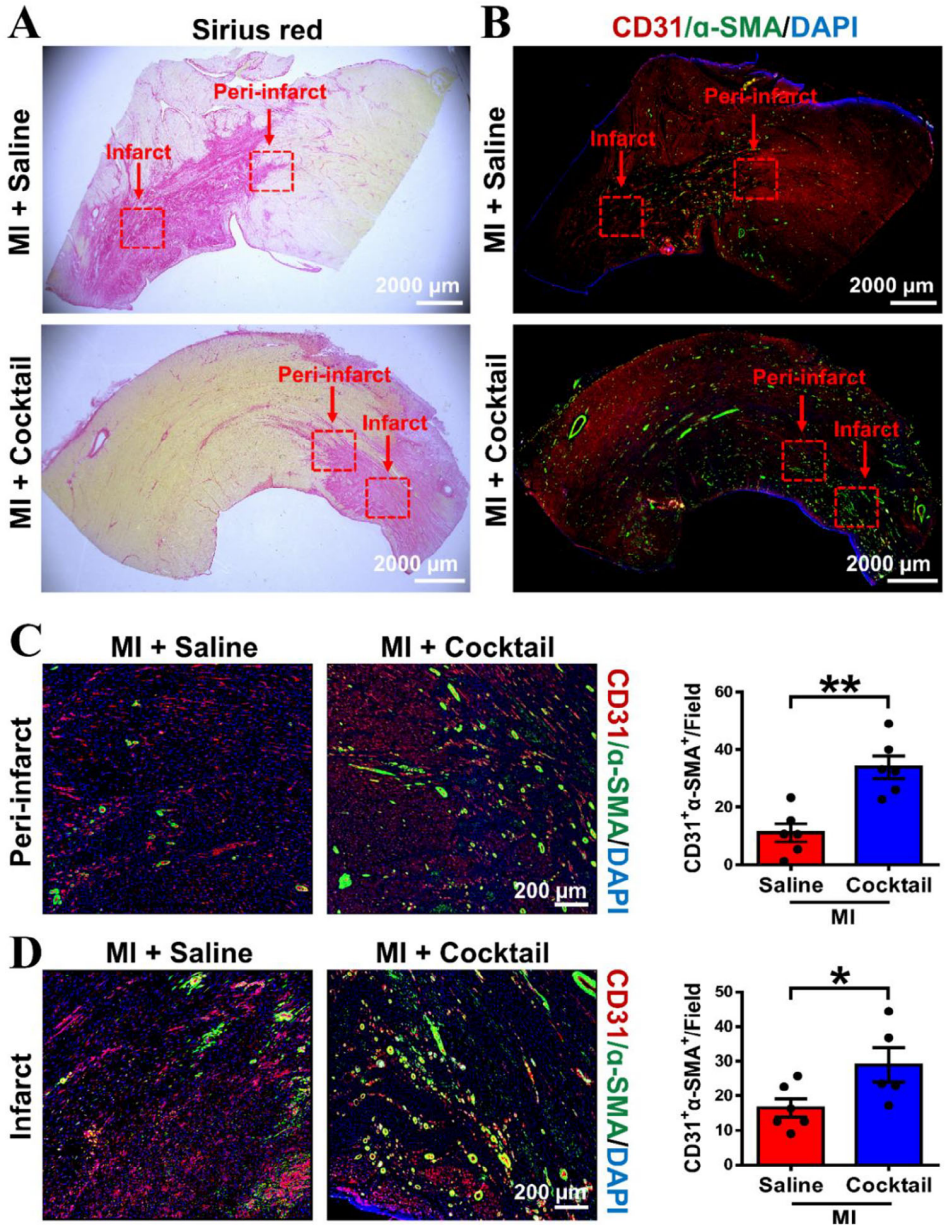
The ion cocktail promotes angiogenesis in Bama minipigs 28 days post‐MI. (A) Representative histological Sirius red staining images (left) and (B) immunofluorescence staining images (right) of CD31 (red), α‐SMA (green) and DAPI (blue) in MI + Saline and MI + Cocktail groups. The red dotted box represents peri‐infarct or infarct regions of the heart. (C) Representative immunofluorescence staining images (magnified from the red dotted box of peri‐infarct areas in (A) and the quantification of CD31^+^α‐SMA^+^ vessels per field in peri‐infarct areas (*n* = 6). (D) Representative immunofluorescence staining images (magnified from the red dotted box of infarct areas in (A) and the quantification of CD31^+^α‐SMA^+^ vessels per field in infarct areas (*n* = 6 for MI + Saline, and *n* = 5 for MI + Cocktail). *p < 0.05 or **p < 0.01.

Next, we also assessed the pro‐angiogenetic effect of ion cocktail by performing immunofluorescence staining of CD31 (an endothelial cell‐specific marker) and α‐SMA on heart tissue sections. Before immunofluorescence staining, the Sirius red staining was conducted to distinguish normal, and infarct areas of cardiac sections from MI + Saline and MI + Cocktail groups, where the infarct area with more collagen deposition was stained with purple while the normal area with invisible collagen was stained with yellow (Figure 5A). The immunofluorescence staining images of CD31 and α‐SMA in peri‐infarct and infarct areas were enlarged, and corresponding quantification results were presented in Figure 5B–D, respectively. It is clear to see that the number of CD31^+^α‐SMA^+^ vessels was significantly higher in MI + Cocktail group as compared to MI + Saline group in both peri‐infarct areas. These comprehensive data suggested that the ion cocktail significantly promoted angiogenesis in both peri‐infarct and infarct areas, thereby reducing infarct size and ultimately restoring cardiac function after MI of Bama minipigs.

### 2.5 The toxicity, bio‐distribution and metabolism analysis of silicate, Sr and Cu ions of the ion cocktail therapy

The critical issue for the clinical application of ion cocktail is the toxicity issue. Thus, we conducted histopathological analyses of livers, spleen, lungs and kidneys in rats and Bama minipigs 28 days post‐MI. As shown in Figure S5A,B, the ion cocktail treatment did not cause obvious structural abnormalities in these organs of both Bama minipig and rat. In addition, we also evaluated the influence of ion cocktail on the liver and kidney function at day28 using automated biochemical analyzer. In addition, we also evaluated the influence of ion cocktail on the liver and kidney function at day28 using automated biochemical analyzer. The result showed that aspartate aminotransferase (ASTL), creatinine (CREA) and blood urea nitrogen (BUN) in rat serum at day 28 were comparable between MI + Saline and MI + ion cocktail groups with no significant differences, which indicated that intravenous injection of the ion cocktail did not affect the function of liver and kidney (Table S3, Supporting Information). To further confirm the biosafety of ion cocktail, we conducted the blood compatibility of the ion cocktail. The result showed that similar to the saline control, the ion cocktail did not cause the hemolysis, while the positive control (ddH_2_O) causes the severe hemolysis (Figure S6A,B). In addition, biochemical testing was performed including prothrombin time (PT) and activated partial thromboplastin time (APTT). The result showed that PT and APTT were comparable among negative control (PPP), saline and ion cocktail groups (Figure S6C,D). Those results indicated that the blood compatibility of the ion cocktail was the same as saline and the ion cocktail has potential for clinical applications. Also, we evaluated the bio‐distribution of Si, Sr and Cu in major organs of rats and Bama minipigs at the end of the animal experiments (14 days after the last injection of ion cocktail). As shown in Figure S7A, the concentrations of Si, Sr or Cu in major organs (liver, lungs, kidneys and aorta) of rats were at the same level in all three groups, indicating no significant accumulation of Si, Sr and Cu in all important organs. It is worth mentioning that Si was relatively evenly distributed in all organs, while more Sr was distributed in aorta and more Cu was distributed in liver and kidneys as compared to other organs. Similar results were shown in Bama minipigs with no significant accumulation of Si, Sr and Cu in all important organs (liver, lungs, kidneys, spleen and aorta) (Figure S7B). To further elucidate the metabolism of Si, Sr and Cu in vivo after injection of ion cocktail in Bama minipigs, we determined the concentrations of Si, Sr and Cu in the serums on 0, 14 and 28 days post‐MI by using ICP‐MS (Figure S8A). The results showed that the concentration of Si (0.16 ± 0.03 µg mL^−1^), Sr (0.18 ± 0.01 µg mL^−1^) and Cu (0.95 ± 0.00 µg mL^−1^) in serums were at the basal level on day 0 (before ion cocktail injection). As expected, the concentrations of the ions in the group with the injection of ion cocktail (MI + Cocktail) for 14 days were much higher than that in the group without the injection of ion cocktail (Sham and MI + Saline) (Si: 11.9 ± 0.3 µg mL^−1^, Sr: 11.62 ± 0.06 µg mL^−1^ and Cu: 12.07 ± 0.64 µg mL^−1^). However, after stopping the injection of ion cocktail, the concentrations of all these ions were recovered to the normal level (Cu) or near the normal level (Si and Sr). Furthermore, we analyzed the metabolism of the Si, Sr and Cu in feces and urine on 1, 7, 14, 21 and 28 day post‐MI, respectively. As shown in Figure S8B,C, the concentration of Si, Sr and Cu in urine and feces was maintained at a higher level during the injection period (1−14 day) and gradually decreased to the normal level in the next 14 days, indicating that ions were mainly excreted through urine and fences metabolism.

### 2.6 The effect of single ions on endothelial cell angiogenesis, macrophage polarization and cardiomyocyte apoptosis

**FIGURE 6:**
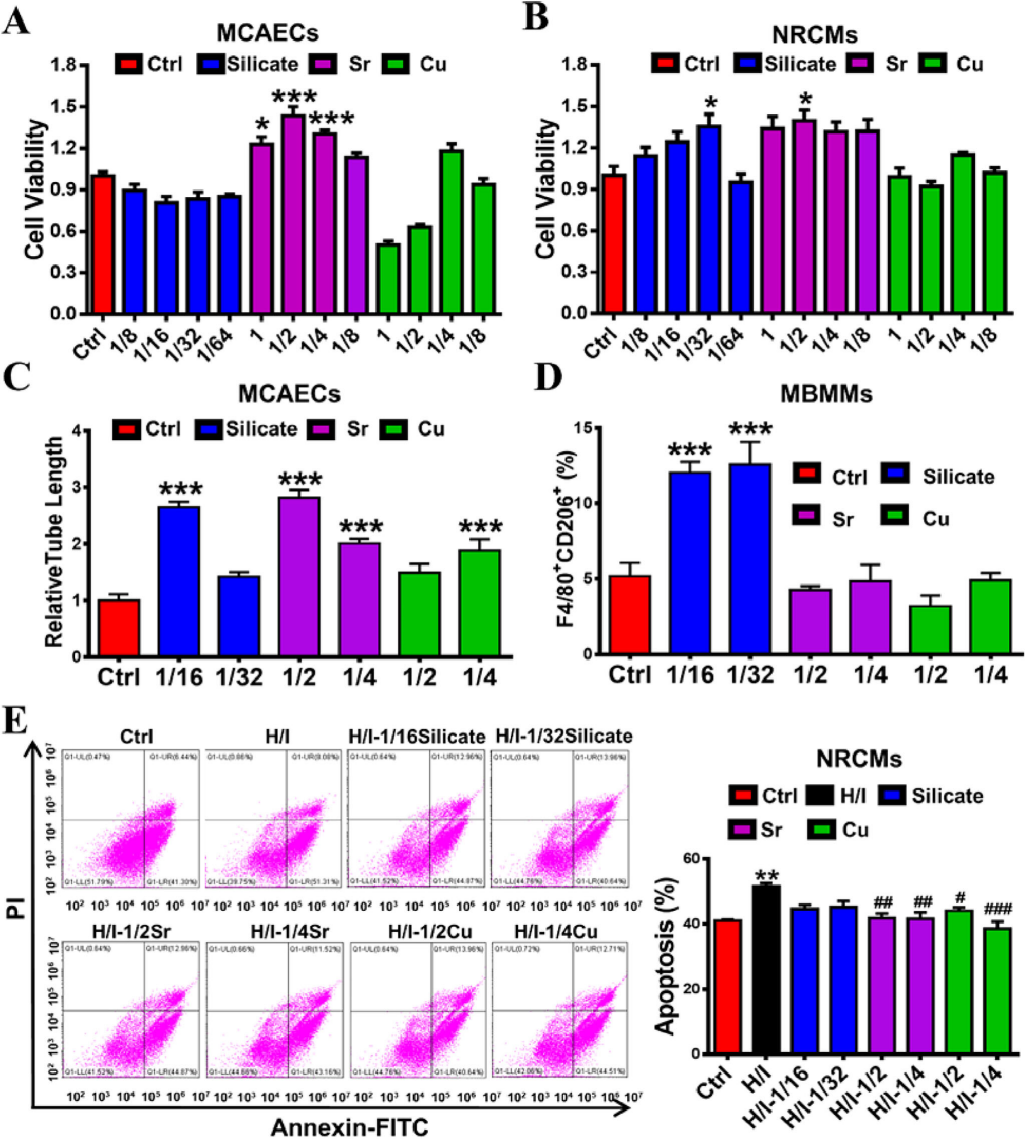
The comparison of single ions (silicate, Sr or Cu) on MCAEC angiogenesis, and MBMM polarization and NRCM apoptosis. (A) The viability of MCAECs treated with different concentrations of single ions for 48 h using the CCK‐8 assay (*n* = 6 for Ctrl, Si and Sr; *n* = 3 for Cu). (B) The viability of NRCMs treated with different concentrations of single ions for 48 h using the CCK‐8 assay (*n* = 5 for Ctrl, Sr (1 and 1/2); *n* = 6 for Si, Sr (1/4 and 1/8) and Cu). (C) The quantification of angiogenesis (presented as relative tube length) in cultured MCAECs treated with different concentrations of single ions for 48 h (*n* = 4 for 1/16Si, 1/4 Sr and 1/2 Cu; *n* = 5 for Ctrl, 1/32Si, 1/2 Sr and 1/4 Cu). (D) The quantitative flow cytometric analysis of MBMM polarization (presented as the percentage of CD206^+^F4/80^+^ cells) treated with different concentrations of single ions for 48 h using phenotypic markers F4/80 (M0) and CD206 (M2) (*n* = 4). (E) Representative flow cytometric images and quantitative analysis of NRCM apoptosis (presented as the percentage of Annexin V^+^ cells) treated with different concentrations of single ions for 48 h after culturing under hypoxia/ischemia condition (H/I) (*n* = 3 for Ctrl; *n* = 4 for Si, Sr and Cu). *p < 0.05, **p < 0.01 or ***p < 0.001 versus Ctrl. ^#^ *p* < 0.05, ^##^ *p* < 0.01 or ^###^ *p* < 0.001 versus H/I. MCAECs: mouse coronary artery endothelial cells. MBMMs: mouse bone marrow‐derived macrophages. NRCMs: neonatal rat cardiac myocytes.

To determine the mechanism of the ion cocktail on the treatment of MI, we compared the biological effects of individual (silicate, Sr and Cu) ions on angiogenesis, immunoregulation and myocardial regeneration using mouse coronary artery endothelial cell (MCAECs), mouse bone marrow‐derived macrophage (MBMMs) and neonatal rat cardiomyocyte (NRCMs), respectively. The cell culture media containing different concentrations of silicate (1/8, 1/16, 1/32, 1/64), Sr (1, 1/2, 1/4, 1/8) or Cu (1, 1/2, 1/4, 1/8) ions were prepared and ion concentrations were measured by inductively coupled plasma mass spectrometry (ICP‐MS) (Table S4). Then, the viability of MCAECs and NRCMs treated with different concentrations of individual ions for 48 h was assessed using CCK‐8 assay. The results showed that different types of ions had different stimulatory effects on the activity of MCAECs and NRCMs in different concentration ranges (Figure 6A,B). For MCAECs, only Sr showed strong stimulation in the concentration range of 1‐1/4 dilutions, but silicate or Cu ions did not. For NRCMs, silicate and Sr ions displayed a stimulatory effect on cells at certain concentrations but not Cu ions. Although Cu ions did not significantly affect the viability of both types of cells, it seems to favor cell growth at the 1/4 concentrations. Therefore, we selected the highest stimulation concentrations of silicate (1/16 and 1/32), Sr (1/2 and 1/4) and Cu (1/2 and 1/4) ions for further cell experiments.

We then systematically compared the effect of the individual (silicate, Sr and Cu) ions on MCAEC angiogenesis, MBMM polarization, and NRCM apoptosis. Firstly, the tube formation assay was conducted to evaluate the effect of individual ions on angiogenesis of MCAECs. As shown in Figure 1, 6, 1/16silicate, 1/2Sr, 1/4Sr and 1/4Cu groups significantly promoted capillary‐like tube formation of MCAECs after culturing for 48 h, as compared to the Ctrl group. Specifically, the 1/2Sr group promoted more capillary‐like tube formation than any other group, indicating 1/2Sr group had the best pro‐angiogenic effect. Then, the flow cytometric analysis with staining of phenotypic markers F4/80 (M0) and CD206 (M2) was conducted to evaluate the effects of individual ions on MBMM polarization to M2 type, which is a pro‐healing phenotype that promotes cardiac tissue repair.^[^ ^12^ ^]^ The representative images and quantitative results were shown in Figure S9 and Figure 6D, respectively. Notably, the percentage of M2 type (CD206^+^F4/80^+^) MBMMs with the treatment of silicate ions (1/16silicate: 12.04 ± 0.73%; 1/32silicate: 12.58 ± 1.50%) for 48 h was about twofold higher than that in Ctrl group (5.18 ± 0.88%). However, the Sr and Cu exhibited a slight but no significant inhibition effect on M2‐type polarization as compared with Ctrl group. Finally, the effect of the individual ions on the protection of cardiomyocytes from hypoxia/ischemia (H/I) injury was evaluated by flow cytometry. As shown in Figure 6E, the percentage of apoptotic NRCMs increased from 41.07 ± 0.36% to 51.57 ± 0.98% when cultured under H/I condition for 8 h, while treatment with individual ions resulted in a decrease to 44.60 ± 1.32% (1/16silicate), 45.18 ± 1.89% (1/32silicate), 41.78 ± 1.39% (1/2Sr), 41.66 ± 1.90% (1/4Sr), 44.05 ± 1.10% (1/2Cu) and 38.55 ± 2.27% (1/4Cu), respectively. It is worth noting that Cu ions, especially the 1/4Cu group, showed the best anti‐apoptotic effect as compared to silicate and Sr ion groups. Interestingly, each ions have a specific activity to preferentially stimulate different type of cells and different cellular behaviors, Sr ions (1/2Sr) revealed the best activity for MCAEC angiogenesis while silicate ions (1/32silicate) showed the highest activity to induce MBMM polarization, and Cu ions (1/4Cu) showed the best beneficial effect on the regulation of NRCM apoptosis. Based on these findings, the ion cocktail was prepared with a final concentration of 1/32silicate, 1/2Sr and 1/4Cu in follow‐up experiments.

### 2.7 The synergetic effects of ion cocktail on the angiogenesis of MCAECs, M2 polarization of MBMMs and apoptosis of NRCMs

**FIGURE 7:**
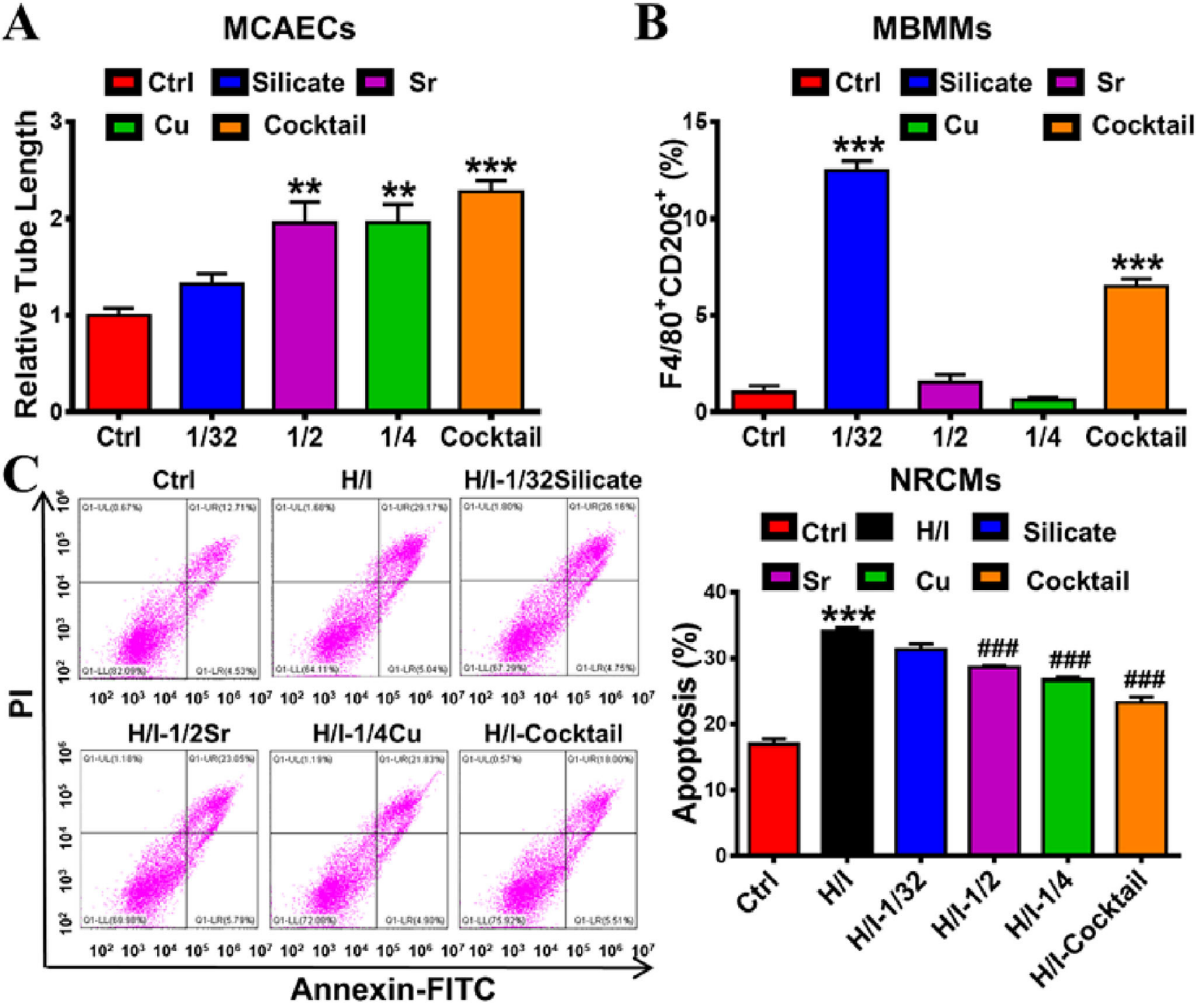
The ion cocktail shows enhanced cellular regulation ability including promoting the angiogenesis of MCAECs, the M2 polarization of MBMMs and inhibiting the apoptosis of NRCMs. (A) The quantification of angiogenesis (presented as relative tube length) in cultured MCAECs treated with single ions and the ion cocktail for 48 h (*n* = 5 for Ctrl, Si and ion cocktail; *n* = 4 for Sr and Cu). (B) The quantitative flow cytometric analysis of MBMM polarization (presented as the percentage of CD206^+^F4/80^+^ cells) treated with single ions and the ion cocktail for 48 h using phenotypic markers F4/80 (M0) and CD206 (M2) (*n* = 4 for Ctrl, Si and Sr; *n* = 3 for Cu and ion cocktail). (C) Representative flow cytometric images and quantitative analysis of NRCM apoptosis (presented as the percentage of Annexin V^+^ cells) treated with single ions or the ion cocktail for 48 h after culturing under hypoxia/ischemia condition (H/I) (*n* = 3 for Ctrl or *n* = 4 for Si, Sr, Cu and ion cocktail). **p < 0.01 or ***p < 0.001 versus Ctrl. ^###^ *p* < 0.001 versus H/I.

After determining the specific activity of different single ions, the most important issue is to verify the synergistic effect of different ion combinations on MCAEC angiogenesis, MBMM polarization to M2 and NRCM apoptosis, which is key to the effectiveness of ion cocktail therapy. Therefore, we compared the pro‐M2 polarization effect of the ion cocktail with the individual ions (1/32silicate, 1/2Sr and 1/4Cu). Interestingly, we observed that the ion cocktail promoted more capillary‐like tube formation of MCAECs than that in groups of 1/32silicate, 1/2Sr or 1/4Cu (Figure 7A). To further clarify the underlying mechanisms of how ion cocktail promotes angiogenesis, we analyzed classical angiogenesis‐related gene signaling pathways, including phosphatidylinositol 3 kinase (PI3K), protein kinase B (AKT), HIF1α and vascular endothelial growth factor (VEGF). The mRNA expression of PI3K, AKT and HIF1α was comparable while VEGF was upregulated in MCAECs after the treatment of ion cocktail compared with single Sr ions (Figure S10A). However, the protein expression of AKT and HIF1α was increased in both Sr ions and ion cocktail groups, although PI3K was unchanged (Figure S10B). These results indicated that both Sr ions and ion cocktail could regulate protein expression but not gene transcription of AKT and HIF1α. To further determine whether the ion cocktail could promote angiogenesis by regulating AKT‐HIF1α axis, the AKT inhibitor AZD5363 was used to block AKT‐HIF1α pathway (Figure S10C). The results showed that, after AZD5363 treatment, the stimulation of protein expression of HIF1α by ion cocktail was indeed significantly inhibited, which indicates that the ion cocktail promotes the angiogenesis of MCAECs possibly by regulating AKT‐HIF1α‐VEGF signaling pathway. In addition, the flow cytometric analysis exhibited that the ion cocktail significantly promoted MBMMs polarization to M2, as the percentage of CD206^+^F4/80^+^ sub‐population (M2 phenotype) in MBMMs was increased from 1.03 ± 0.33% to 6.49 ± 0.40%, which was much higher than 1/2Sr or 1/4Cu group but lower than 1/32silicate group (Figure S11; Figure 7B). This is expected as 1/4Cu displayed a negative effect on the polarization of macrophage towards M2 type in single ions experiments. Besides, as shown in Figure 7C, the apoptotic NRCMs in ion cocktail group showed a sharp decrease from 34.05 ± 0.56% (H/I group) to 23.28 ± 0.84%, which was less than that in the 1/32 silicate (31.27 ± 0.90%), 1/2Sr (28.64 ± 0.22%) or 1/4Cu (26.65 ± 0.49%) group. Anyway, all these results demonstrated that ion cocktail had the best comprehensive performance in inhibiting apoptosis of NRCMs, stimulating angiogenesis of MCAECs and promoting polarization of MBMMs to M2 type.

## 3 DISCUSSIONS

Myocardial infarction (MI) is associated with high mortality and disability. Although different therapeutic strategies such as pharmacological therapy, gene therapy and cell therapy show great potential for the treatment of MI, limitations still exist. In our previous study, we have shown that silicate ions have therapeutic effect on MI, but this single ion treatment may not be able to solve the problem with both cardiac structural and electrical remodeling after MI. Here, we reported an ion cocktail therapy by using three different types of ions with different specific biological functions to regulate different cells and pathological processes, and the unique therapeutic effect of the ion cocktail was proofed by both rats and Bama minipigs MI models. We found that the ion cocktail significantly improved the cardiac function of Bama minipigs after MI and reduced the infarct size by 55%. This protective effect was better than other therapeutic strategies such as stem cell‐derived cardiac cell patches,^[^ ^13^ ^]^ and AAV9‐Salvador gene therapy.^[^ ^11^ ^]^ The therapeutic effect of the ion cocktail may be attributed to the accumulation of the ion combination in the myocardium at the early stage of ion injection. Our previous studies have shown that intramyocardial injection of Sr or intravenous injection of silicate ion caused a sharp increase in ion at day 1 and 7, which indicated that the therapeutic effect on the injured heart is caused by the ion injection.^[^ ^3^, ^4^, ^14^ ^]^ Based on our previous findings, it is assumed that therapeutic effect of the ion cocktail may attribute to the accumulation of ion combination at infarct heart after injection, but further systematic analysis of the ion distribution at different time points in different area of the infarct heart is required for understanding the effect of the ion cocktail.

At present, the therapeutic strategies for MI mainly include regulating cardiac structural (e.g. inhibition of collagen deposition, cardiomyocyte hypertrophy and scar tissue formation) or electrical remodeling (e.g. reduction of APD90, conduction velocity, AP dispersion and Ca^2+^ disorder). Some treatments such as adeno‐related virus 9 (AAV9)‐miR‐199a gene therapy and human pluripotent stem cell‐derived cardiomyocytes therapy have shown great potential for attenuating the cardiac structural remodeling of MI by reducing infarct area and collagen deposition, but they may also cause cardiac electrical instability and increase the risk of arrhythmia, indicating that these strategies only improve cardiac structural remodeling after MI, but may not decrease the patient's risk of death.^[^ ^2^, ^15^ ^]^ Li et al., have shown that small extracellular vesicles containing miR‐486‐5p have a good effect on attenuating cardiac structural remodeling, but not on electrical remodeling post‐MI.^[^ ^2b^ ^]^ Although some injectable and conductive cardiac patches were shown to attenuate both the structural and electrical remodeling after MI, they only increase the conduction of electrical signals with limited therapeutic effect.^[^ ^16^ ^]^ In the present study, we demonstrated that ion cocktail therapy effectively attenuated both electrical and structural remodeling post‐MI. Compared with current MI treatment strategies that only target the conduction of electrical signals, ion cocktail therapy reduces the incidence of EAD/DAD, electrical heterogeneity, and VF of the heart, which strongly confirms the positive effect of ion cocktail therapy on electrical remodeling. Although the ion cocktail did not alter the APD90 or CaD90, it significantly reduced the dispersion of APD90 or CaD90. These results are correlated with the findings in previous studies which showed that IQR was increased in MI patients or mice even if the APD90/CaD90 was not altered.^[^ ^9^, ^17^ ^]^ In addition to the IQR measurement, we introduced the difference value (DV) of both APD90 and CaD90 between the infarct areas and peri‐infarct/normal areas as a new measurement for the electrical uniformity of the infarct heart. Our results showed a significant increase of the DV of APD90 and CaD90 for MI heart as compared to the Sham group, while the ion cocktail treatment significantly decreased the DV of both APD90 and CaD90, indicating a reduced heterogeneity of cardiac electrophysiology in infarct heart. Moreover, heterogeneously distributed infarcted tissue in MI results in the initiation and maintenance of electrical instability, which leads to arrhythmia.^[^ ^18^ ^]^ Indeed, our results imply the possibility of “ion cocktail therapy” for recovering cardiac electrical instability and alleviating ventricular fibrillation after MI. The strong therapeutic effect of the ion cocktail on cardiac electrophysiology may be attributed to the bioactive ions, which can largely influence cardiac electrophysiological characteristics through ion channels and the effect may be controlled by adjusting different types and concentrations of ions, in which specific ion types and concentrations have different roles and functions through different mechanisms. Sr ions have been found to prolong the action potential at the concentration of 2.7 mmol L^−1^.^[^ ^6^ ^]^ Sr and calcium (Ca) ions share some common ion channels and could be transported by the Na^+^‐Ca^2+^ exchanger and bind to cardiac troponin C to induce cardiac ventricular myocyte contraction.^[^ ^19^ ^]^ The Ca^2+^‐permeable non‐selective cation channel transient receptor potential vanilloid‐3 (TRPV3) could also mediate Sr ion influx.^[^ ^20^ ^]^ Cu is a blocker of the Ca^2+^‐permeable cationic channel (TRPM2).^[^ ^7^ ^]^ Cu could induce sodium (Na) ion transport/potassium chloride (KCl) extrusion via Na^+^/H^+^ exchanger and K^+^/Cl^−^ volume‐sensitive channels, respectively.^[^ ^21^ ^]^ Although silicate ion channels have not been reported in mammals so far, we speculate that the anion chloride channels, which play a profound role in the development of ischemic heart diseases,^[^ ^22^ ^]^ may mediate the intracellular intake of anionic silicate ions.

Timely repair of the vascular network in infarct area is an important strategy in the treatment of acute MI.^[^ ^23^ ^]^ Many studies have revealed that silicate ions have activity in the stimulation of EC proliferation, and up‐regulation of different pro‐angiogenic factors such as vascular endothelial growth factor and its receptor, resulting in improved wound healing,^[^ ^24^ ^]^ myocardial regeneration^[^ ^3^, ^25^ ^]^ and vascularization of different tissues.^[^ ^26^ ^]^ Similar to silicate ions, pro‐angiogenic effects have also been observed in Sr and Cu ions.^[^ ^27^ ^]^ For example, Sr‐containing albumin hydrogels promoted vascularization of infarcted areas in mice.^[^ ^4a^ ^]^ Cu content in ischemic myocardium was found to be dramatically reduced after MI injury, accompanied by the inhibition of vascular neogenesis,^[^ ^4b^ ^]^ indicating the vital role of Cu ions in vascularization of the heart after MI. More interestingly, our study demonstrated that silicate, Sr and Cu ions modulated the activity of MCAECs and promoted their angiogenic properties in vitro and ion cocktail resulted in a significantly higher angiogenic effect than each type of ions.

Promotion macrophage polarization toward the M2 phenotype is an important strategy for the treatment of MI.^[^ ^28^ ^]^ A previous study reported that silicate ions (1.2−1.8 µg mL^−1^) promoted THP‐1 polarization toward the M2 phenotype, and our previous results also revealed that silicate ions (4.8 and 2.4 µg mL^−1^) significantly stimulated M2 polarization of MBMMs. In addition, a previous study showed that the extract of Sr‐containing bioactive glass microspheres (6.22 µg mL^−1^) promoted macrophage M2 polarization.^[^ ^29^ ^]^ However, the immunomodulatory effect of Cu ions on macrophage polarization is controversial. Cu ions in a concentration of 3.25 µg mL^−1^ were reported to promote M1 polarization, thereby enhancing osteogenic and antibacterial effects,^[^ ^30^ ^]^ while a different study reported that Cu ions in a concentration range of 0.781–25 mg mL^−1^ promoted M2 polarization of macrophages,^[^ ^31^ ^]^ indicating a concentration‐dependent role of Cu ions in regulating macrophage polarization, and in the low concentration range it mainly induces M1 polarization. Although in the present study, Cu ions in the concentration of 1.3 µg mL^−1^ and 0.6 µg mL^−1^ revealed a slight inhibitory effect on M2 polarization, its combination with Sr and silicate ions together in ion cocktail still revealed high activity to stimulate M2 polarization of macrophages indicating a balanced functional effect of the ion combination.

Loss of cardiomyocytes after MI is the pathological basis for heart failure.^[^ ^32^ ^]^ The present study found that all three ions inhibited cardiomyocyte apoptosis. Surprisingly, we found that Cu ions (1.3 µg mL^−1^ and 0.65 µg mL^−1^) strongly inhibited cardiomyocyte apoptosis, and its anti‐apoptotic effect was even stronger than silicate and Sr ions. Such anti‐apoptotic effects may account for the fact that Cu ions function as an essential catalytic cofactor for various enzymes including superoxide dismutase and cytochrome c oxidase, which are related to mitochondrial function and cell apoptosis.^[^ ^33^ ^]^ Notably, for the first time, we demonstrated that the combination of silicate, Sr and Cu ions had a significantly higher inhibitory effect on cardiomyocyte apoptosis than the individual ions.

By further analyzing the underlying mechanisms we found that ion cocktail could activate the AKT‐HIFα‐VEGF signaling pathway, which is correlated well with the literature that Sr ions could promote angiogenesis by activating AKT‐HIF1α‐VEGF signaling pathway during bone regeneration.^[^ ^34^ ^]^ Interestingly, our study, which different from previous reports, revealed that Sr ion and ion cocktail do not affect the gene transcription but activate protein translation of AKT and HIF1α to promote angiogenesis of endothelial cells. Considering that silicate ions could activate mitogen‐activated protein kinase (MAPK)‐nuclear factor kappa‐B (NF‐κB) signaling pathway,^[^ ^35^ ^]^ the possible mechanism of silicate ions for activation of M2 polarized macrophages may be through the regulation of NF‐κB pathway.^[^ ^36^ ^]^ Based on the fact that Sr ions can fully activate the calcium‐sensing receptor (CaSR)‐phospholipase (PLC)‐NF‐κB^[^ ^37^ ^]^ while Cu ions can activate the ATP7A‐microRNA5b‐NF‐κB signaling pathway,^[^ ^38^ ^]^ we speculate that the Sr and Cu ions may also regulate macrophage M2 polarization through NF‐κB pathway.^[^ ^30^ ^]^ In addition, it is known that Cu ions may regulate the activity of cytochrome oxidase (CCO) and Fas‐Fas‐associated death domain (FADD), thereby affecting cell apoptosis,^[^ ^33^, ^39^ ^]^ whereas the regulation of cell apoptosis by Sr and silicate ions may be achieved through the TGFβ‐smad2/3 signaling pathway^[^ ^40^ ^]^ and extracellular signal‐regulated kinase (ERK)‐p38 mitogen activated protein kinase (p38MAPK),^[^ ^3b^ ^]^ respectively. Based on our results and literature analysis, each type of ion within the cocktail functions as the main activator for one specific regulation, and the other two ions act synergistically to further enhance the regulatory effect (Figure S12).

For clinical applications, safety issue needs to be considered. In previous studies, we have analyzed the bio‐distribution of silicate and Sr ions in single ion‐based material treatment, and proofed primary safety of single ion treatment.^[^ ^3^, ^4^ ^]^ In this study, we analyzed the ion distribution in important organs including liver, lung, kidney, spleen, and aorta, and found no significant ion accumulation in these organs. In addition, we analyzed aspartate aminotransferase (ASTL), creatinine (CREA) and blood urea nitrogen (BUN) in rat serum, which did not show any significant difference as compared with the control group. This result suggests that the ion cocktail treatment did not affect liver and kidney function. Furthermore, we tested the blood compatibility of the cocktail and further confirmed the safety of the ion combination. Generally speaking, the composition of the ion cocktail in this study is simple, mainly metabolized from urine, and there is no obvious ion accumulation in tissues and organs, so it might be safer than traditional drugs. Regarding the function of the ion combination, the therapeutic effect of the cocktail treatment seems also better than some other therapeutic strategies reported in the literature such as stem cell‐derived cardiac cell patches,^[^ ^13^ ^]^ and AAV9‐Salvador gene therapy.^[^ ^11^ ^]^ To some extent, the advantages of the ion cocktail does not limit to the dual‐reduction of both structural and electrical remodeling of the heart after MI but may also include the avoidance of some disadvantages related to other strategies such as the immunogenicity of antibody drugs, the invasiveness of myocardial patch therapy and the safety of AAV‐mediated gene therapy. Therefore, considering both therapeutic effect and safety, the cocktail strategy might have great clinical translational potential, although systematic studies still required to further elucidate the mechanisms of the ion combination treatment and long‐term safety.

In summary, our results demonstrated that the ion cocktail treatment could attenuate both structural remodeling and electrical remodeling of the heart after MI. The mechanisms of the ion cocktail for regulation of structural remodeling can be explained by the synergistic regulatory effects of different ion combinations on cellular behaviors including the angiogenic activity of endothelial cells, macrophage polarization and cardiomyocyte apoptosis. Furthermore, the anion and cation combination of the ion cocktail revealed strong activity in regulating cardiac electrophysiology and significantly reduced the electrophysiology heterogeneity of infarct heart possibly through different ion channels, which is critical for reducing the risk of arrhythmia. Our study provides a new therapeutic strategy with high effectiveness and safety for the treatment of MI with clinical translation potential.

## 4 METHODS

### 4.1 Preparation of the single ion solutions (silicate, Sr or Cu) and ion cocktail solution

For in vivo animal experiments, the CS bioceramics were extracted in the saline and then the strontium chloride or copper chloride powder were dissolved in the CS bioceramic extract to prepare the ion cocktail with the final concentration of 105 µg mL^−1^ for silicate, 250 µg mL^−1^ for Sr and 20 µg mL^−1^ for Cu.

For in vitro cell experiment, a high concentration stock solution with a concentration of 1250 µg mL^−1^ for Sr ion or 100 µg mL^−1^ for Cu ion was prepared by dissolving strontium chloride or copper chloride powder (Macklin Biochemical Co., Ltd., Shanghai, China) in ddH_2_O. Then, a low concentration stock solution with a concentration of 25 µg mL^−1^ for Sr ion or 2.0 µg mL^−1^ for Cu ion was prepared by dilution of the high concentration stock solution in DMEM. The stock solution of silicate ions with a concentration of about 105 µg mL^−1^ were prepared by dissolution of CS bioceramics in saline for 24 h. Then, the Sr or Cu stock solution was diluted in DMEM to the ratio of 1/2, 1/4 and 1/8, while the stock solution of silicate ion was diluted in the DMEM to the ratio of 1/8, 1/16, 1/32 and 1/64. The optimal concentrations of silicate (diluted to the ratio of 1/32), Sr (diluted to the ratio of 1/2) and Cu (diluted to the ratio of 1/4) for cell activity were determined using cell activity assay. Finally, the ion cocktail for cell culture experiments was prepared by mixing three low concentration stock solutions with the final concentration of silicate for 1/32 dilution, Sr for 1/2 dilution and Cu for 1/4 dilution, respectively (Figure S13).

### 4.2 Rat myocardial infarction (MI) model and the ion cocktail treatment

Specific pathogen‐free adult male rats weighing 350–450 g were obtained from SPF Biotechnology Co., Ltd. (Beijing, China). The animal protocol and experimental procedures were approved by the Animal Research and Ethics Committee of Scope Research Institute of Electrophysiology Co. Ltd. (Approval Issue No. SGLL22022301). For the rat MI model, the experiment was performed according to the previously described protocol.^[^ ^41^ ^]^ Briefly, rats were anesthetized with pentobarbital sodium (80 mg kg^−1^). Mechanical ventilation was then achieved by connecting the endotracheal tube to a scientific ventilator (ZS‐MV‐HX, Beijing, China), cycling at 80 breaths per minute and a tidal volume of 1.2 mL per 100 g body weight. Sequentially, surgery was operated on, and the left anterior descending (LAD) artery was ligated with a slipknot using sterile 6/0 silk sutures. In sham control rats, the procedure was identical except for the ligation of LAD. Ischemia was confirmed by visual observation of the left ventricle (LV) wall turning pale and the presence of ST‐Segment elevation on the electrocardiogram (BL‐420N, Chengdu, China). Then, the ribs were drawn together with sterile 2/0 silk sutures and the skin was closed with sterile 6/0 silk sutures. After surgery, the ion cocktail‐containing saline or saline control was intravenously injected 8 times every other day. The injection volume of the ion cocktail varied slightly according to the weight of the rats (3 mL kg^−1^). The experimental groups were defined as Sham, MI + Saline and MI + Cocktail, respectively.

### 4.3 Optical mapping and data analysis

For the optical mapping of rats with MI, the experimental procedure was as follows: rats were euthanized with isoflurane followed by an intraperitoneal injection of heparin (120 IU) at 28 days post‐MI, and then hearts were quickly taken out. The residual lung tissues were removed in pre‐cooling Krebs–Henseleit (KH) solutions containing (mm): 119, NaCl; 25, NaHCO_3_; 4, KCl; 1.2, KH_2_PO_4_; 1, MgCl_2_.6H_2_O; 1.8, CaCl_2_.2H_2_O; 10, d‐glucose (pH = 7.4, filled with 95% O_2_ and 5% CO_2_). Next, the aorta was cannulated and connected to the Langendorf‐perfused system (SGL, SCOPE, China) with a flow rate of 10 mL min^−1^ and at 37 ± 0.5°C in circulated KH solution (100 mL). Adding 300 µL blebbistatin (an excitation‐contraction uncoupler, 1 mg mL^−1^ in DMSO), 50 µL Pluronic F127 (a membrane pore‐forming agent), 100 µL Rhod‐2 AM (an intracellular Ca^2+^ indicator, 1 mg mL^−1^ in DMSO) and 100 µL RH237 (a voltage‐sensitive dye, 1 mg mL^−1^ in DMSO) in circulated KH solution (100 mL). Emission light was then produced with two LED light sources at 530 nm and bandpass filtered from 511 to 551 nm (LEDC‐2001, MappingLab, UK). The longer wavelength, containing Vm signals, was long bypass‐filtered at 770 nm and the shorter wavelength, containing Ca^2+^ signals, was long bypass‐filtered between 574 nm and 606 nm. The emitted fluorescence signals were recorded with CMOS cameras (OMS‐PCIE‐2002, Mapping Lab, UK) at a sampling rate of 1000 Hz from a 6.4 × 6.4 mm field of view (64 × 64 pixels). Optical signals were spatially aligned and processed using a Gaussian spatial filter (3 × 3 pixels). The repolarization time at 90% return to baseline was calculated as APD90, while Ca^2+^ transient duration at 90% recovery to baseline was calculated as CaD90. The ventricular fibrillation was induced by the programmed stimulation with an S1S1 interval (30 times, S1 stimuli at 150 ms). Optical mapping data was performed using a commercially available analysis program (OMapScope4.0, MappingLab, UK).^[^ ^42^ ^]^ Electrophysiological parameters were recorded at baseline (5 Hz pacing) or with the treatment of isoprenaline (1 µm, 7 Hz pacing). Optical mapping data was recorded every 5 min following treatment and some hearts that could not maintain normal rhythms were abandoned.

### 4.4 Bama minipig MI model and the ion cocktail treatment

Specific male Bama minipigs weighing 20–30 kg were obtained from the Jian Lin breeding farm (Harbin, China) for this study. The animal protocol and experimental procedures were approved by the Animal Research and Ethics Committee of the Second Affiliated Hospital of Harbin Medical University (Approval Issue No. SYDW2022‐023). The method to establish Bama minipig MI model was performed according to the previously described protocol.^[^ ^43^ ^]^ Briefly, Bama minipigs were fasted for 12 h before surgery and then injected with atropine (0.05 mg kg^−1^) to reduce oral secretions. After being anesthetized by propofol (1–2 mg kg^−1^), Bama minipigs were transferred to the operating table with an ear intravenous drip of Zoletil (2–4 mg kg^−1^). The mechanical ventilation was then achieved by connecting the endotracheal tube to a scientific anesthetic system (AM811, Beijing, China), cycling at 20 breaths per minute, a tidal volume of 10–12 mL per kg body weight, and suction/respiration ratio of 1:2. Meanwhile, anesthesia was maintained with 1−2% isoflurane and 0.5−1.5 L min^−1^ O_2_ during the entire operation. Pulse oxygen saturation, heart rate and blood pressure were monitored using a vital signs monitor (Mindray IPM2, Shenzhen, China). Subsequently, the chest near the left thoracotomy (between the IV rib) was opened and the pericardium was scratched. LAD coronary artery located at 1/3 of the anterior descending coronary artery near the apex was ligated with sterile 5/0 silk sutures, followed by drawing the sternum together with sterile 3/0 silk sutures and closing the skin with sterile 0/0 silk sutures. Lidocaine and nitroglycerin diluted in saline were sprayed on the surface before ligation to prevent ventricular fibrillation. Ischemia was confirmed by visual observation of the LV wall turning pale and the presence of ST elevations on the electrocardiogram (Mindray IPM2, Shenzhen, China). Antibiotics were fed regularly to prevent infection. After surgery, about 25 mL of the ion cocktail‐containing saline or saline control was intravenously injected 8 times every other day. The injection volume of the ion cocktail varies slightly according to the weight of the Bama minipigs (0.7 mL kg^−1^). The experimental groups were defined as Sham, MI + Saline and MI + Cocktail, respectively.

### 4.5 Transthoracic echocardiography of Bama minipigs cardiac function

Transthoracic echocardiography was performed on the anesthetized Bama minipigs using GE Vivid E9 ultrasound system (GE Medical Systems, Milwaukee, WI, USA) just before MI, 0 (immediately after MI), 14 and 28 days post‐MI. Two‐dimensional short‐axis views were visualized at the levels of apex (apical level). Left ventricle fractional shortening (LVFS) and left ventricle ejection fraction (LVEF) were measured in M‐mode tracings at the apical level.

### 4.6 Histology and immunofluorescence evaluation

The rat hearts were rinsed with PBS three times after the electrophysiological experiments finished and then fixed with 4% paraformaldehyde (PFA) for 12 h. followed by dehydration and paraffin‐embedding. Samples in the middle of the ventricle were sectioned with a thickness of 5 µm for Masson's trichrome and immunofluorescence staining. The infarcted size was measured by the ratio of total fibrosis area to the entire area of LV using ImageJ software (National Institutes of Health, USA) based on Masson's trichrome staining. For immunofluorescence staining, the obtained sections were deparaffinized and rehydrated, treated with 3% hydrogen peroxide, and heated for antigen retrieval in 10 mm citrate acid buffer before being blocked with 5% BSA for 60 min. Then the primary antibodies against CD31 (Abcam, ab28364, 1:200) and cTnI (Proteintech, 21652‐1‐AP, 1:200) were incubated overnight at 4°C followed by incubation with the secondary antibody of Cy3‐conjugated Affinipure Goat Anti‐Rabbit IgG (H + L) (Proteintech, SA00009‐2, 1:400) and Fluorescein (FITC)‐conjugated Affinipure Goat Anti‐Mouse IgG (H + L) (Proteintech, SA00003‐1, 1:400) for 120 min at room temperature. Sequentially, cell nuclear was stained with 4′6‐diamidino‐2‐phenylindole (DAPI, Solarbio, C0065) for 10 min. The fluorescence signals were monitored by a Nikon A1 confocal microscopy (Nikon, Japan) and vessel density in different areas (normal, peri‐infarct and infarct) was analyzed by calculating the capillary density (CD31 positive).

Morphometric and histological analysis of samples from Bama minipigs were consistent with rats with a minor modification. Briefly, after 28 days post‐MI, Bama minipigs were euthanized, and the hearts were quickly collected and shocked in PBS to remove the excess blood. Ventricles were sectioned from apex to base into six to eight transverse slices and each slice was photographed. The degree of infarction was measured by manual tracing with the ratio of total infarct area to the entire area of LV using ImageJ software (National Institutes of Health, USA). The method was conducted as previously described.^[^ ^44^ ^]^ Next, the samples containing both of peri‐infarct area and the infarct area were collected, fixed with 4% PFA for 12 h, embedded in paraffin, and sectioned at a thickness of 5 µm for Sirius red and immunofluorescence staining. The Sirius red staining was performed using a commercial kit (Solarbio, G1471). The primary antibodies against CD31 (Servicebio, GB13063, 1:200), and α‐SMA (Abcam, ab7817, 1:400), and the secondary antibody of Cy3‐conjugated Affinipure Goat Anti‐Rabbit IgG (H + L) (Proteintech, SA00009‐2, 1:400), Alexa Fluor 647‐conjugated Affinipure Donkey Anti‐Goat IgG (Abcam, ab150131, 1:400) and Fluorescein (FITC)‐conjugated Affinipure Goat Anti‐Mouse IgG (H + L) (Proteintech, SA00003‐1, 1:400) were used for immunofluorescence staining. For evaluation of the toxicity of ion cocktail to rats and pigs, the organs of both rats and pigs (livers, spleen, lungs and kidneys) were isolated, fixed with 4% PFA for 12 h, and then embedded with paraffin to prepare sections. Five‐micrometer serial sections were cut and then stained with HE (Solarbio, G1120). The images were captured for morphological analysis.

### 4.7 The blood compatibility assay

Rabbit blood was collected in the anticoagulant tube. 1 mL of whole blood was diluted with 5 mL of saline solution and 100 µL ion cocktail was added to 2 mL of saline solution. After that, 100 µL of diluted whole blood was added to the diluted ion cocktail solution. The same amount of diluted whole blood was added to ddH_2_O and saline as positive control and negative control, respectively. All samples were incubated at 37°C for 2 h, centrifuged at 1500 rpm for 5 min, and photographed. 200 µL supernatant was added to 96‐well plate and absorbance was measured at 540 nm by an enzyme labeling instrument. The hemolysis rate is calculated according to the following formula: Hemolysis (%) = (sample abs540−negative control abs540)/(positive control abs540−negative control abs540)×100%. Whole blood PT and APTT were determined by an automatic coagulation analyzer. Rabbit whole blood was collected into an anticoagulant tube and centrifuged at 3000 rpm for 15 min to obtain the supernatant, namely platelet‐poor plasma (PPP). 25 µL ion cocktail or saline was absorbed and incubated with 250 µL PPP for 20 min. The CaCl_2_ solution, APTT and PT reagents are placed in the initial position of the coagulation analyzer and wait for the test temperature to be reached. PPP was used as the blank control group for APTT and PT experiments.

### 4.8 Biochemical analysis in rat serum

Serum from rats was collected on day 28 after MI. The absolute values of aspartate aminotransferase (ASTL), creatinine (CREA) and BUN in rat serum were measured using an automated biochemical analyzer (Roche COBAS8000).

### 4.9 The metabolism and distribution of Silicate, Sr and Cu

The ion (Silicate, Sr and Cu) metabolism and distribution in serum (collected at 0, 14 and 28 days post‐MI), urine and feces (collected at 0, 7, 14, 21, 24 and 28 days post‐MI) of Bama minipigs, as well as in major organs (collected at 28 days post‐MI) of rats (including liver, lungs, kidneys and aorta) and Bama minipigs (including liver, lungs, kidneys, spleen and aorta) were evaluated by using the inductively coupled plasma mass spectrometer (ICP‐MS, Agilent 7850, USA). Briefly, the tissues or feces were dissolved in aqua regia for about 5 days and then filtered with a 0.22 µm membrane. The serum or urine was diluted with deionized water 2–5 times before detection.

### 4.10 Cell isolation and culture

The mouse coronary artery endothelial cells (MCAECs) were purchased from Mingzhou Biotechnology Co., Ltd. (Zhejiang, China) and cultured in high glucose DMEM supplemented with 10% FBS and 1% P/S in a humid atmosphere of 5% CO_2_ at 37°C. The identity of MCAECs was assessed by immunofluorescence staining of marker CD31 (Abcam, ab28364). The cells from passages 3 to 10 were used in this study.

The primary mouse bone marrow‐derived macrophages (MBMMs) were isolated from 2‐mouth‐old male mice and differentiated in high glucose DMEM supplemented with 10% FBS, 20% L929‐conditioned medium, and 1% P/S using a standard protocol.^[^ ^45^ ^]^ Then cells were cultured in high glucose DMEM supplemented with 10% FBS and 1% P/S in a humid atmosphere of 5% CO_2_ at 37°C. The primary MBMMs were identified by immunofluorescence staining of marker F4/80 (Santa Cruz, sc‐377009) and MBMMs were used without passage.

Neonatal rat cardiomyocytes (NRCMs) were prepared as previously described with a slight modification.^[^ ^46^ ^]^ Briefly Sprague–Dawley (SD) rats at 1−3 day‐old was purchased from Jiesjie Laboratory Animal Co., Ltd. (Shanghai, China) for heart collection. Then, the ventricle tissues in heart tissues were dissected into pieces of 2 mm^3^ and followed by digestion within 0.25% trypsin at 4°C for 12 h. Sequentially, the pieces of the heart were enzymatically digested in collagenase II solution (1 mg mL^−1^) for about 3 h. Finally, the ventricular cardiomyocytes were separated from fibroblasts by differential plating and then cultured in high glucose DMEM supplemented with 10% fetal bovine serum (FBS), 1% penicillin/streptomycin (P/S) and 0.003% 5‐Bromo‐2′‐deoxyuridine in a humid atmosphere of 5% CO_2_ at 37°C. NRCMs were identified by immunofluorescence staining of marker Cardiac Troponin I (cTnI, Proteintech, 21652‐1‐AP) and used without passage.

### 4.11 Cell viability detection

Before adding a single ion (silicate, Sr or Cu)‐containing medium, the MCAECs were seeded on the 96‐well plates at a density of 3 × 10^3^ cells cm^−2^, while the NRCMs were seeded on the 96‐well plates at a density of 4.5 × 10^4^ cells cm^−2^ and cultured in normal media for 48 h and cultured in normal media for 12 h. Then, the culture media were replaced by a medium with different concentrations of silicate (1/8, 1/16, 1/32 and 1/64), Sr (1, 1/2, 1/4 and 1/8) or Cu (1, 1/2, 1/4 and 1/8) ion and cultured for 48 h. The cell viability was measured by a CCK8 assay kit (Dojindo, Kumamoto, Japan) according to the manufacturer's instructions. The absorbance was detected using a microplate reader (Synergy 2, BioTek, USA) at the wavelength of 450 nm.

### 4.12 Tube formation assay

Tube formation assay was conducted as previously described.^[^ ^47^ ^]^ Briefly, the MCAECs were cultured in silicate (1/16 and 1/32), Sr (1/2 and 1/4) Cu (1/2 and 1/4) ion, or the ion cocktail‐containing medium for 48 h. Then, 250 µL of reduced growth factor Matrigel (BD, 356230) was pipetted into 24‐well plates and solidified at 37°C for 30 min. Once the Matrigel was set, different ions‐treated MCAECs (5 × 10^4^ cells cm^−2^) were added in and cultured for 6 h. The formed tube was captured by Orthographic microscope (CKX53, Japan) and the tube length was analyzed using the Image J software with the Angiogenesis Analyzer plugin (National Institutes of Health, USA).

### 4.13 Quantitative real‐time polymerase chain reaction (qPCR) and western blot analysis

qPCR and western blot were analyzed as previously described.^[^ ^36^, ^48^ ^]^ Briefly, cells were treated with single ions or ion cocktail with/absence of AZD5363 (AKT inhibitor, MCE, Cat# HY‐15431) for 48 h. For qPCR, the RNA Rapid Extraction Kit (Cat# 11201es08; Yeasen, Shanghai, China) was used for total RNA extraction. Then, the RNA concentration was measured by an ultra‐micro spectrophotometer (DeNovix, DS‐11, USA). Subsequently, the complementary DNA (cDNA) was synthesized with a cDNA Synthesis kit (Cat# 11141es60; Yeasen, Shanghai, China) after the RNA concentration was quantified. Finally, the quantification of all cDNA was carried out using a LightCycler 480 II (Roche, Swiss) fluorescence real‐time PCR system. The primers of PI3K, AKT, HIF1α and VEGF were listed in Table S5. For western blot, the protein was extracted with IP lysis (Beyotime, Cat# P0013J) buffer and separated by SDS‐PAGE electrophoresis using standard methods. Then, the protein was transferred to PVDF membrane, and immunoblotted with specific primer antibodies overnight at 4°C against PI3K (Proteintech, Cat# 20584‐1‐AP), AKT (Beyotime, Cat# P0013J), HIF1α (Beyotime, Cat# P0013J), β‐Actin (Beyotime, Cat#AF0003) and GAPDH (Beyotime, Cat# AF1891). Next, the proteins were incubated with HRP‐conjugated goatanti‐rabbit or mouse secondary antibody for 1 h at room temperature. Finally, the proteins were visualized with an X‐ray film system (Fujifilm, Japan) or ChemiDoc XRS + System (Bio‐Rad, USA). Protein was quantified by NIH ImageJ software (National Institutes of Health, USA) and the protein expression was normalized to β‐actin or GAPDH.

### 4.14 Flow cytometric analysis

For the analysis of MBMM polarization, PE anti‐mouse F4/80 (BioLegend, 123109) and Brilliant Violet 650 anti‐mouse CD206 (BioLegend, C068C2) antibodies were used. Briefly, more than 1×10^4^ MBMMs were incubated with F4/80 antibodies for 30 min at 4°C and then treated with Cyto‐Fast Fix/Perm (Biolegend, B335149) for cell membrane rupture. Subsequently, a CD206 antibody was used to label MBMMs at 4°C for 40 min. After the unbound antibodies were washed out, the labeled cells were analyzed using a BD FACS Celesta system (CytoFLEX, Beckman Coulter, USA). The percentage of M2‐type macrophages was defined as CD206^+^F4/80^+^ cells gated from single cells.

For the analysis of NRCMs apoptosis, the hypoxia/ischemic (I/H) model of NRCMs was performed as previously described with a gentle modification.^[^ ^49^ ^]^ Briefly, the NRCMs were cultured in the glucose‐free medium for 8 h under an atmosphere of O_2_ less than 0.1% using a multi‐gas incubator (APM‐30DR, Japan). Then, the silicate, Sr, Cu or the ion cocktail‐containing culture medium was replaced to culture NRCMs for 48 h. The Annexin V‐FITC Apoptosis Detection Kit (Beyotime, Shanghai, China) was used to detect the apoptosis of NRCMs. Briefly, ion‐treated cells were re‐suspended in apoptosis‐positive control solution (195 µL) and then mixed with Annexin V‐FITC (5 µL) and propidium iodide (PI, 10 µL), avoiding light for 20 min. Sequentially, the labeled cells were analyzed using a BD FACS Celesta system (CytoFLEX, Beckman Coulter, USA).

### 4.15 Statistical analysis

All assessments were conducted by an investigator who was blinded to the experimental condition and/or treatment group. All data were presented as the mean ± SEM. Statistical analysis was performed using GraphPad Prism7 (Beijing, China). Differences between percentages were assessed by χ2 test or Fisher's exact test. A two‐sided unpaired Student's *t*‐test was used to analyze data between the two groups. Differences were assessed by one‐way analysis of variance (ANOVA) with Tukey's multiple comparison test for multiple groups. A two‐way ANOVA was carried out using the Bonferroni post hoc test for experiments in different groups and at different times. Non‐normally distributed data were analyzed by Mann–Whitney's U test. *p* < 0.05 is considered statistically significant.

## AUTHOR CONTRIBUTIONS

Yumei Que, Jiaxin Shi and Zhaowenbin Zhang contributed equally to this work. Jiang Chang, Chen Yang and Yumei Que designed the experiments. Zhaowenbin Zhang prepared the ion solutions. Yumei Que, Yanxin Zhang, Xin Li, and Xiao Yang performed the in vitro experiments (cell culture and isolation), Yumei Que analyzed the optical and electroanatomic mapping data. Jiaxin Shi, Lu Sun, Hairu Li, Xionghai Qin, Chong Liu, Chang Liu and Shijie Sun performed the Bama minipig study (surgery and echocardiography). Yumei Que and Qishu Jin performed the qPCR, western blot and histology experiments. Yumei Que, Chen Yang, Zhen Zeng and Jiang Chang wrote the manuscript. Jiang Chang, Jiawei Tian, Hai Tian and Chen Yang conceived the conceptual ideas and supervised the study.

## CONFLICT OF INTEREST STATEMENT

The authors declare no conflicts of interest.

## Supporting information

Supporting Information

## Data Availability

The raw/processed data forms part of an ongoing study and may be requested from the authors.
