# Supplementary material for: Ion cocktail therapy for myocardial infarction by synergistic regulation of both structural and electrical remodeling
Source: Exploration (Beijing). 2023 Nov 23;4(3):20230067. doi: 10.1002/EXP.20230067 (PMC11189571; doi:10.1002/EXP.20230067)
Supplement: Supplementary file 1 — Supporting Information [file EXP2-4-20230067-s001.docx]

**Ion cocktail therapy for myocardial infarction by synergistic regulation of both structural and electrical remodeling.**

Yumei Que^1,3,§^, Jiaxin Shi^5,§^, Zhaowenbin Zhang^2,4,§^, Lu Sun^7,8^, Hairu Li^5^, Xionghai Qin^6,8^, Zhen Zeng^1,3^, Xiao Yang^3^, Yanxin Chen^1,3^, Chong Liu^5^, Chang Liu^8^, Shijie Sun^5^, Qishu Jin^1,3^, Yanxin Zhang^3^, Xin Li^3^, Ming Lei^9^, Chen Yang^1,3*^, Hai Tian^6,8*^, Jiawei Tian^5*^, Jiang Chang^1,2,3,4*^

*^1^ Joint Centre of Translational Medicine, the First Affiliated Hospital of Wenzhou Medical University, Wenzhou, Zhejiang 325000, China*

*^2^ Shanghai Institute of Ceramics, Chinese Academy of Sciences (CAS), 1295 Dingxi Road, Shanghai 200050, China*

*^3^ Zhejiang Engineering Research Center for Tissue Repair Materials, Wenzhou Institute, University of CAS, Wenzhou, 325000, China*

*^4^ Center of Materials Science and Optoelectronics Engineering, University of CAS, 19 Yuquan Road, Beijing, 100049, China*

*^5^ Department of Ultrasound, the Second Affiliated Hospital of Harbin Medical University, Harbin, 150081, China.*

*^6^ Department of Cardiovascular surgery, the Second Affiliated Hospital of Harbin Medical University, Harbin, 150086, China.*

*^7^ Department of Cardiovascular Surgery, Peking University Shenzhen Hospital, Shenzhen, 518036, China.*

*^8^ Future Medical Laboratory, the Second Affiliated Hospital of Harbin Medical University, Harbin, 150086, China.*

*^9^ Department of Pharmacology, University of Oxford, Mansfield Road, Oxford OX1 3QT, UK.*

^§^ Equally contributed to this work.

^*^ Corresponding authors.

E-mail addresses: [jchang@mail.sic.ac.cn](mailto:jchang@mail.sic.ac.cn) (Jiang Chang)；[jwtian2004@163.com](mailto:jwtian2004@163.com) (Jiawei Tian); [doctor_tianhai@163.com](mailto:doctor_tianhai@163.com) (Hai Tian); cryangchen@ucas.ac.cn (Chen Yang).

**Supplemental Figures**


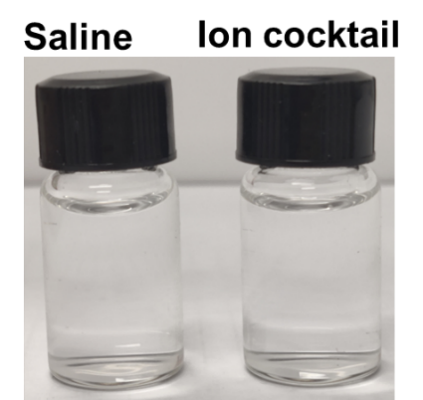


**Figure S1** Representative image of ion cocktail solution diluted in saline for *in vivo* experiments.


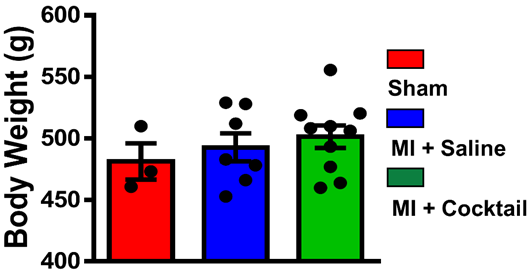


**Figure S2** The effect of the ion cocktail on body weight of rat 28 days post-MI. The quantification of the body weight in different groups. (n=3 for Sham, n=7 for MI + Saline and n=10 for MI + Cocktail).


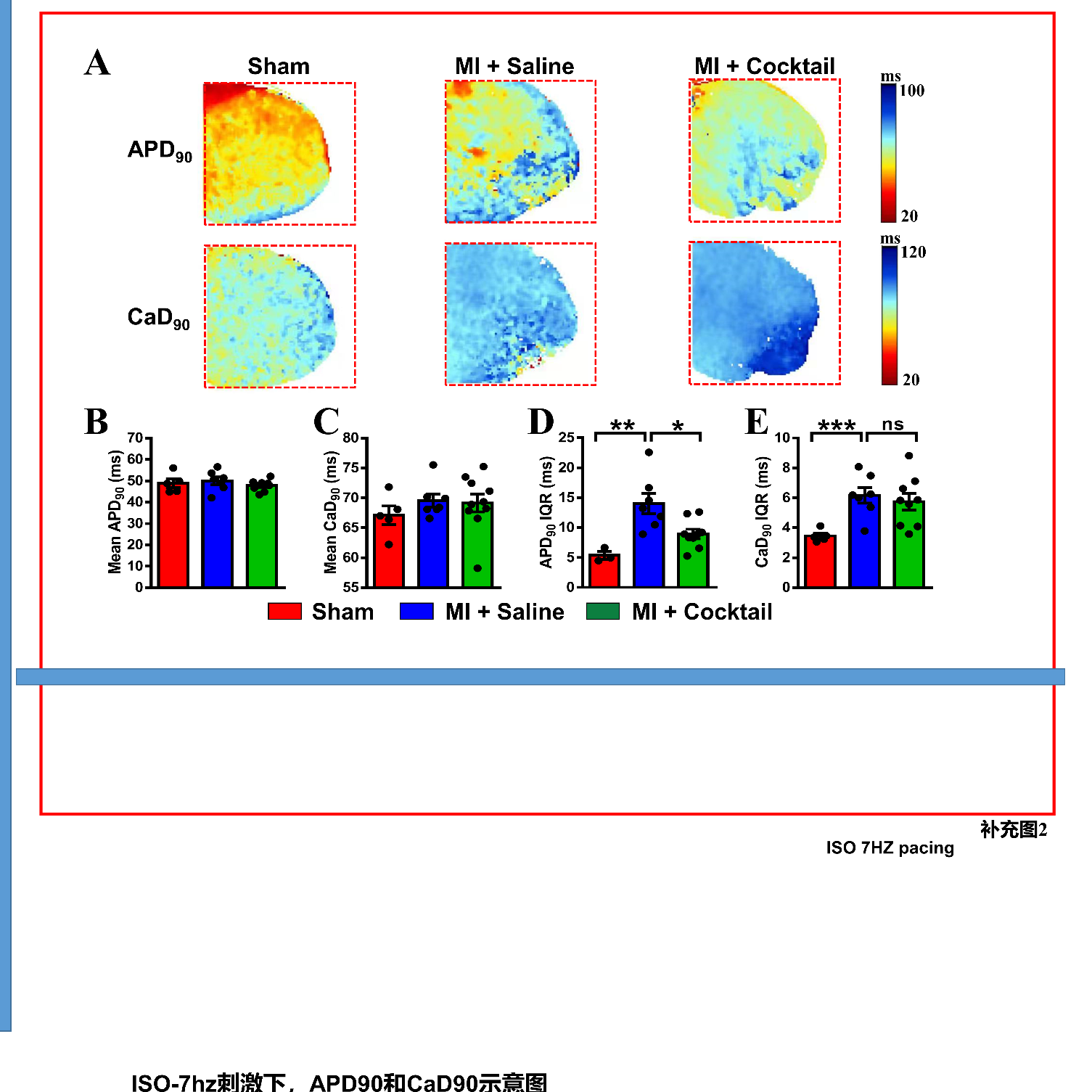


**Figure S3** The ion cocktail limits dispersion of APD90 following ISO treatment in rats 28 days post-MI. **A)** The representative maps of APD90 (top) and CaD90 (bottom). The quantification of **B)** mean APD90; **C)** mean CaD90 (n=5 for Sham, n=7 for MI + Saline and n=10 for MI + Cocktail); **D)** IQR of APD90; **E)** IQR of CaD90 in different groups. (n=3 for Sham, n=7 for MI + Saline and n=9 for MI + Cocktail). **P< 0.01 or ***P< 0.001. ns: not significant. ISO: isoproterenol.


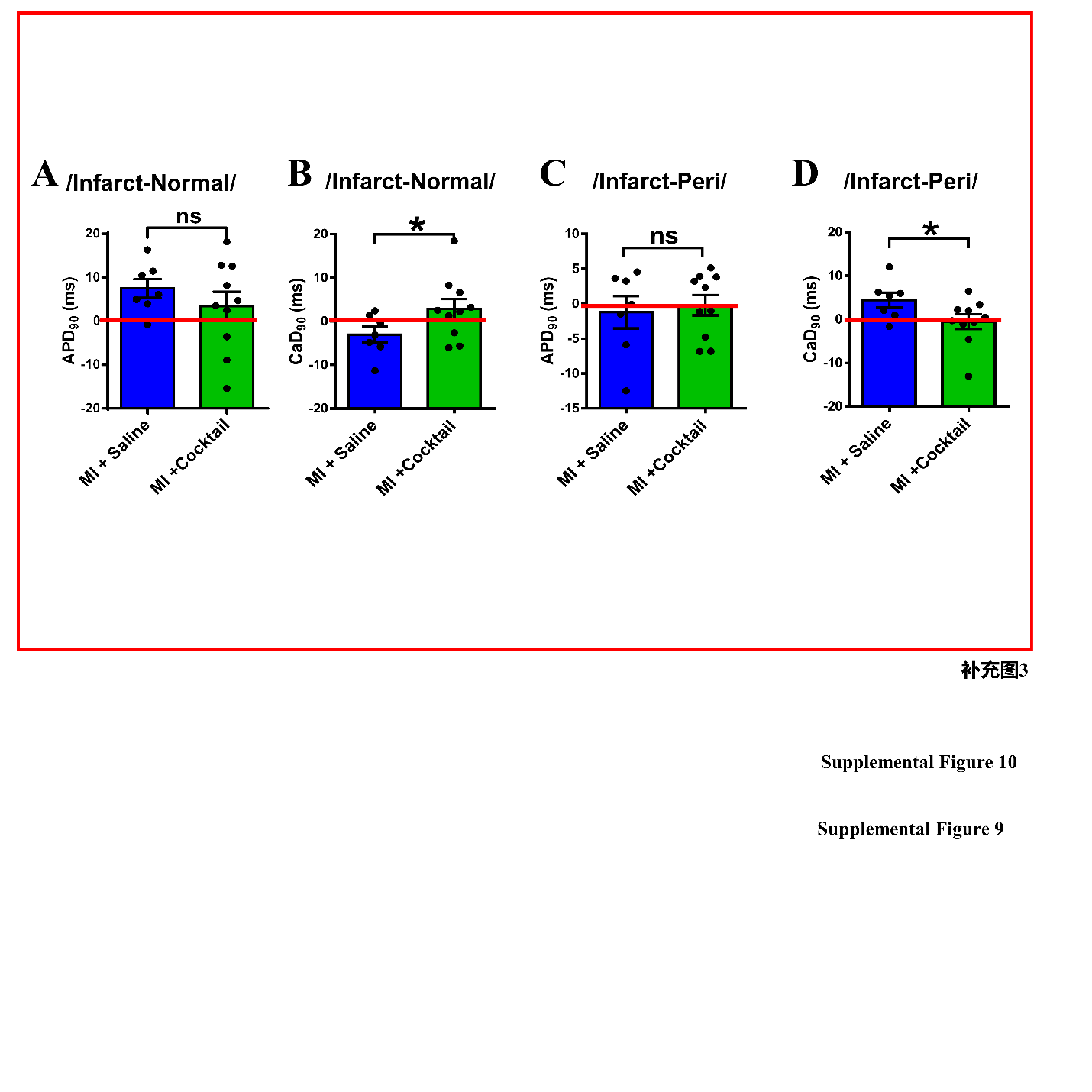


**Figure S4** The ion cocktail decreased uniformity of the cardiac electrophysiology between normal and infarct areas or between infarct and peri-infarct areas in rats after ISO stimulations 28 days post-MI. The quantification of difference value of **A)** APD90 and **B)** CaD90 in infarct and normal areas or **C)** APD90 and **D)** CaD90 infarct and peri-infarct areas in different groups. (n=7 for MI + Saline and n=10 for MI + Cocktail). *P< 0.05. ns: not significant. ISO: isoproterenol.


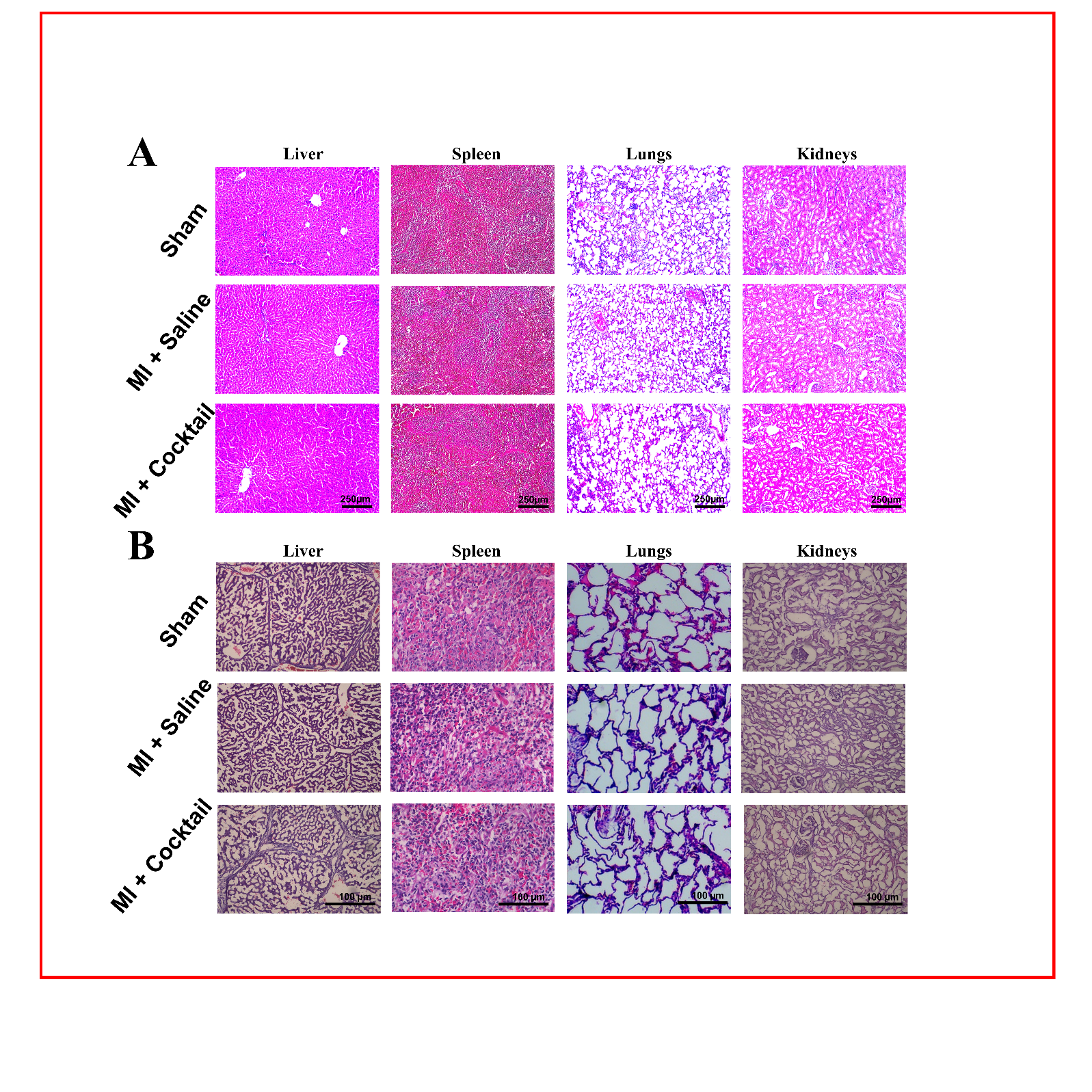


**Figure S5** The ion cocktail has no organ toxicity in rats and Bama minipigs 28 days post-MI. Representative histological HE staining of crucial organs including liver, spleen, lungs and kidneys of **A)** rats and **B)** Bama minipigs. (n=3).


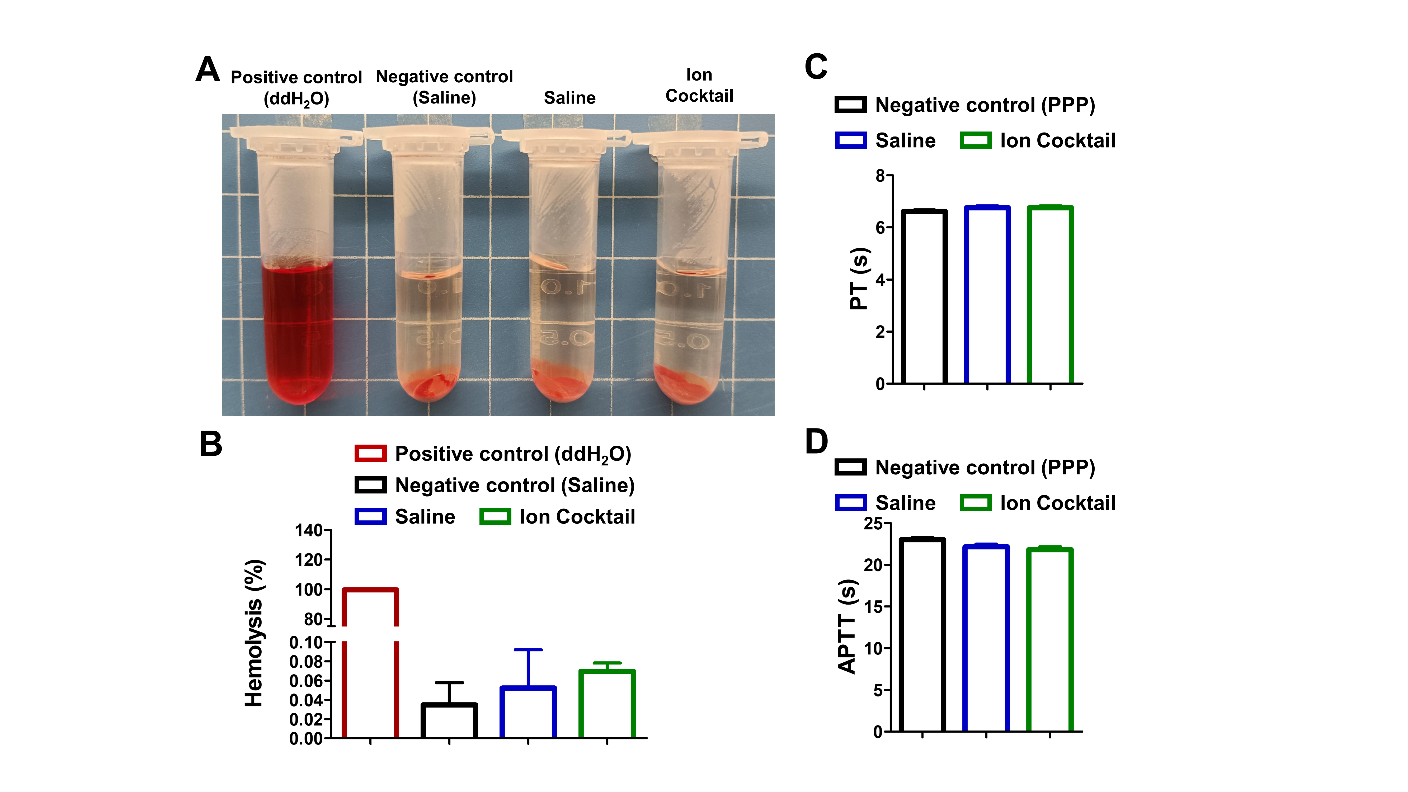


**Figure S6 A)** Representative hemolysis with positive control (ddH_2_O), negative control (saline), saline or ion cocktail. **B)** The quantification of hemolysis with positive control (ddH_2_O), negative control (saline), saline or ion cocktail. (n=3). The quantification of **C)** PT and **D)** APTT among different groups (n=5). Data are presented as mean ± SEM, one-way ANOVA. PPP, platelet-poor plasma.


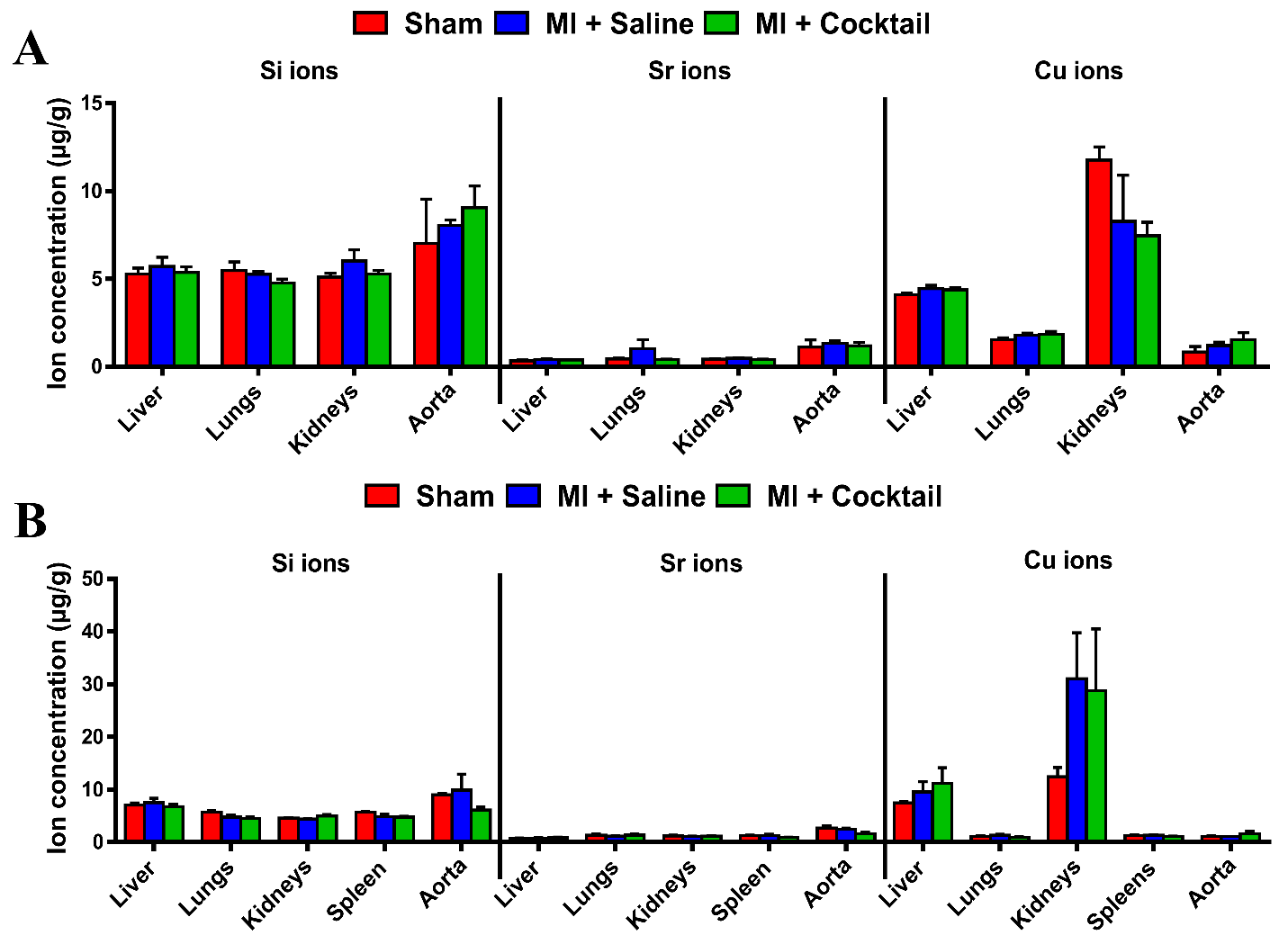


**Figure S7** Bio-distribution of Si, Sr, or Cu after ion cocktail treatment in crucial organs of rats and Bama minipigs 28 days post-MI. **A)** The concentrations of Si, Sr, or Cu in liver, lungs, kidneys, and aorta of rats; **B)** The concentrations of Si, Sr, or Cu ions in liver, lungs, kidneys, spleen, and aorta of Bama minipigs (n=3).


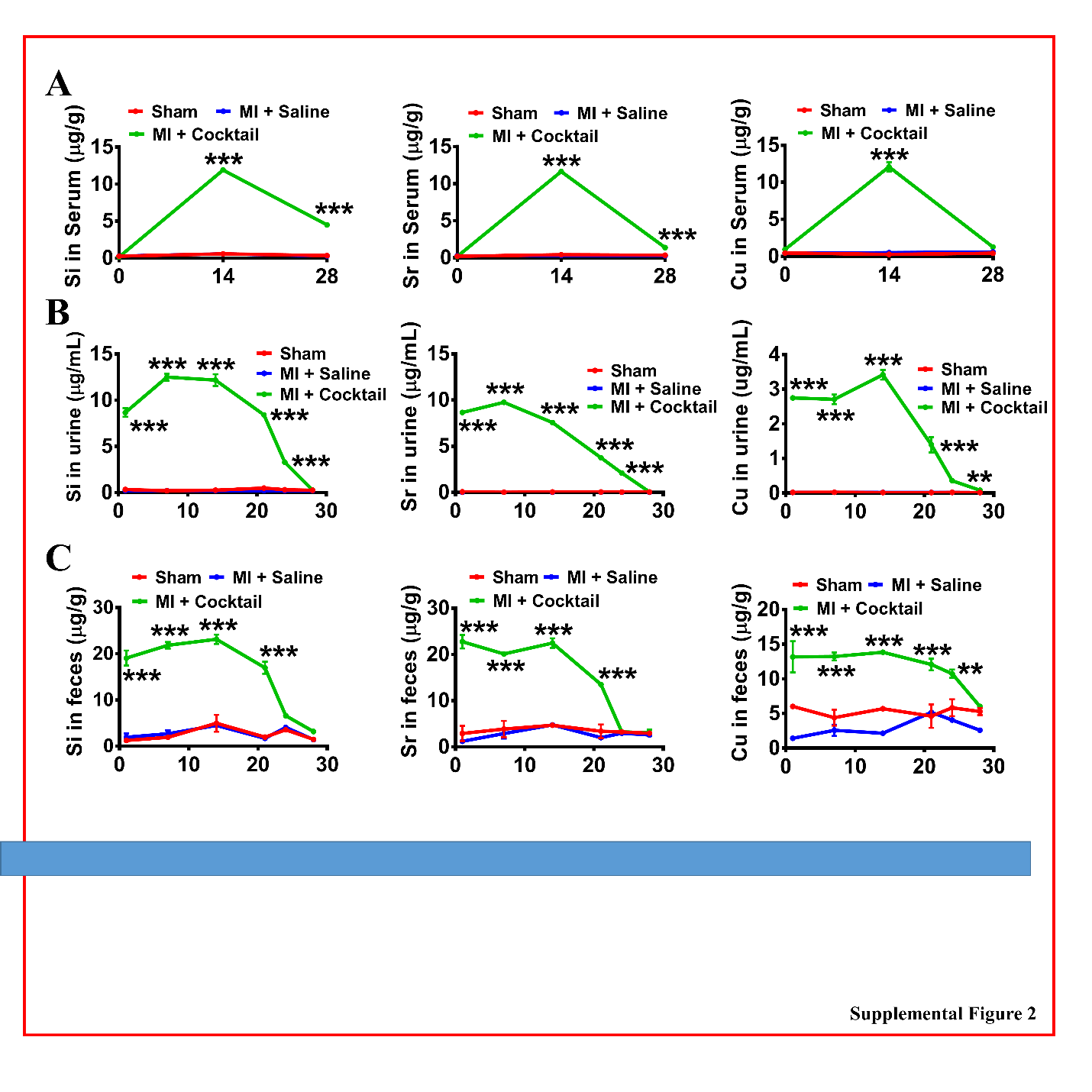


**Figure S8** Metabolism of Si, Sr, or Cu in serum, urine, and feces of Bama minipigs after ion cocktail treatment. **A)** The concentrations of Si, Sr, and Cu ions in serum 0, 14, and 28 days post-MI; The concentrations of Si, Sr, and Cu ions in urine **B)** or feces **C)** at 0, 7, 14, 21, 24, and 28 days post-MI. (n=3). ***P<0.001 or **P<0.01 vs. Sham and MI + Saline.


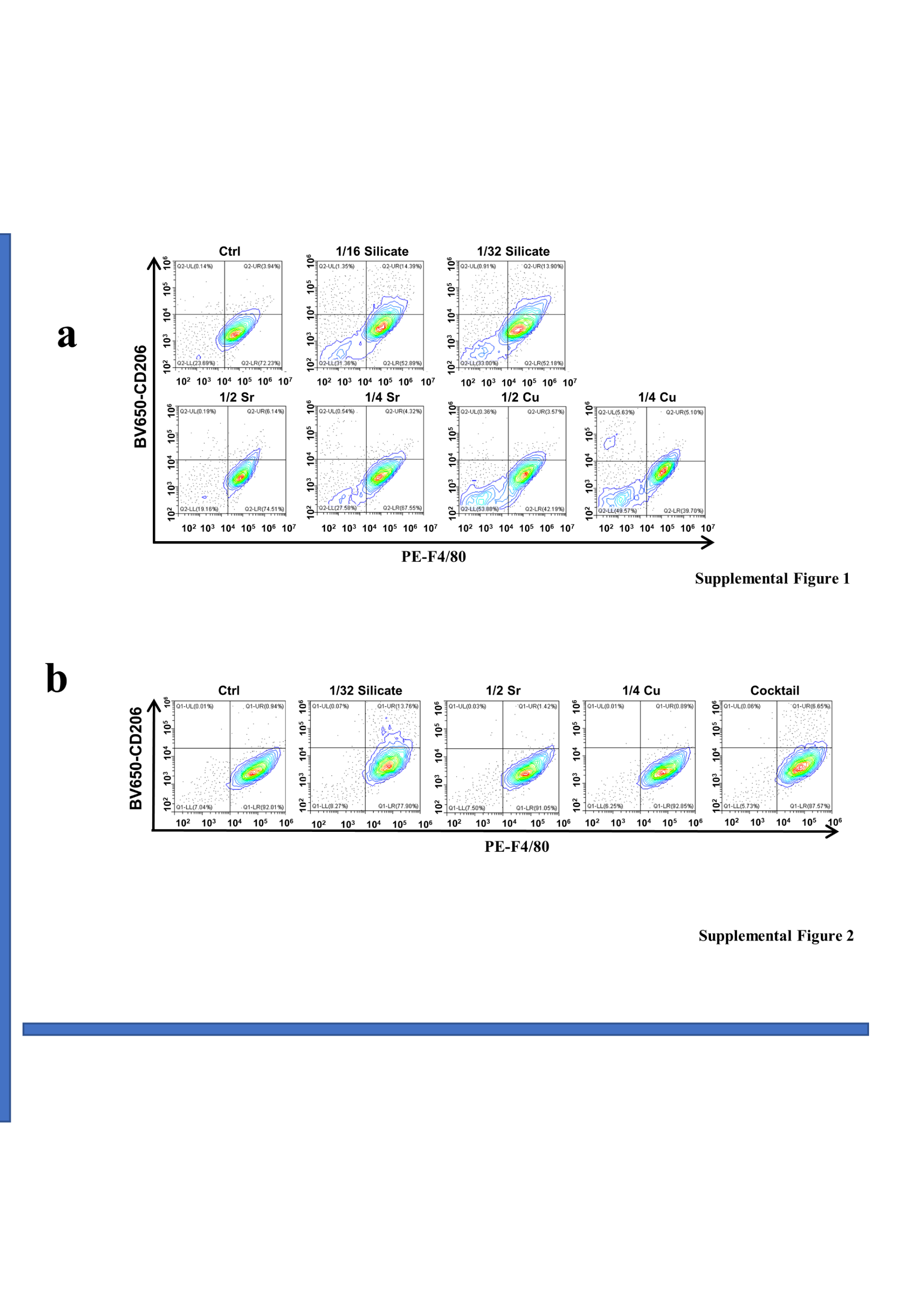


**Figure S9** Representative flow cytometric images of M2-type polarization of MBMM (presented as the percentage of CD206^+^F4/80^+^ cells) treated with different concentrations of single ions (silicate, Sr, or Cu) for 48 h using phenotypic markers F4/80 (M0) and CD206 (M2). Ctrl, control medium


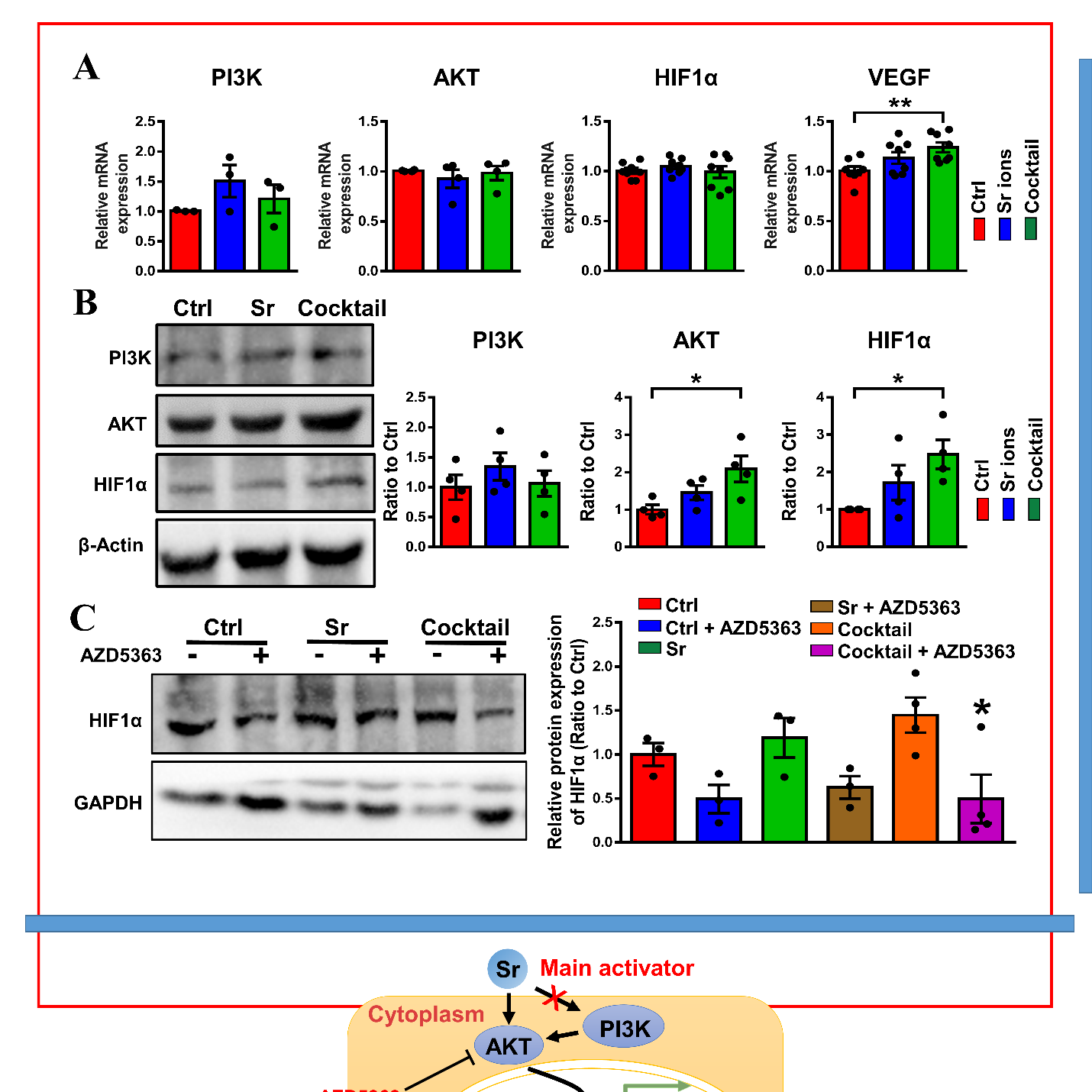


**Figure S10** The mechanisms of the ion cocktail on promoting angiogenesis of MCAECs. **A)** The mRNA expression of PI3K, AKT, HIF1α and VEGF treated with Sr ions or ion cocktail. (n=3 or 6). **B)** The representative WB image and quantification of protein expression of PI3K, AKT and HIF1α in MCAECs treated with Sr ions or ion cocktail. (n=4). **C)** The representative WB image and quantification of protein expression of HIF1α treated with Sr ions or ion cocktail with/absence of AZD5363 (AKT inhibitor) (n=3 or 4). *P< 0.05.


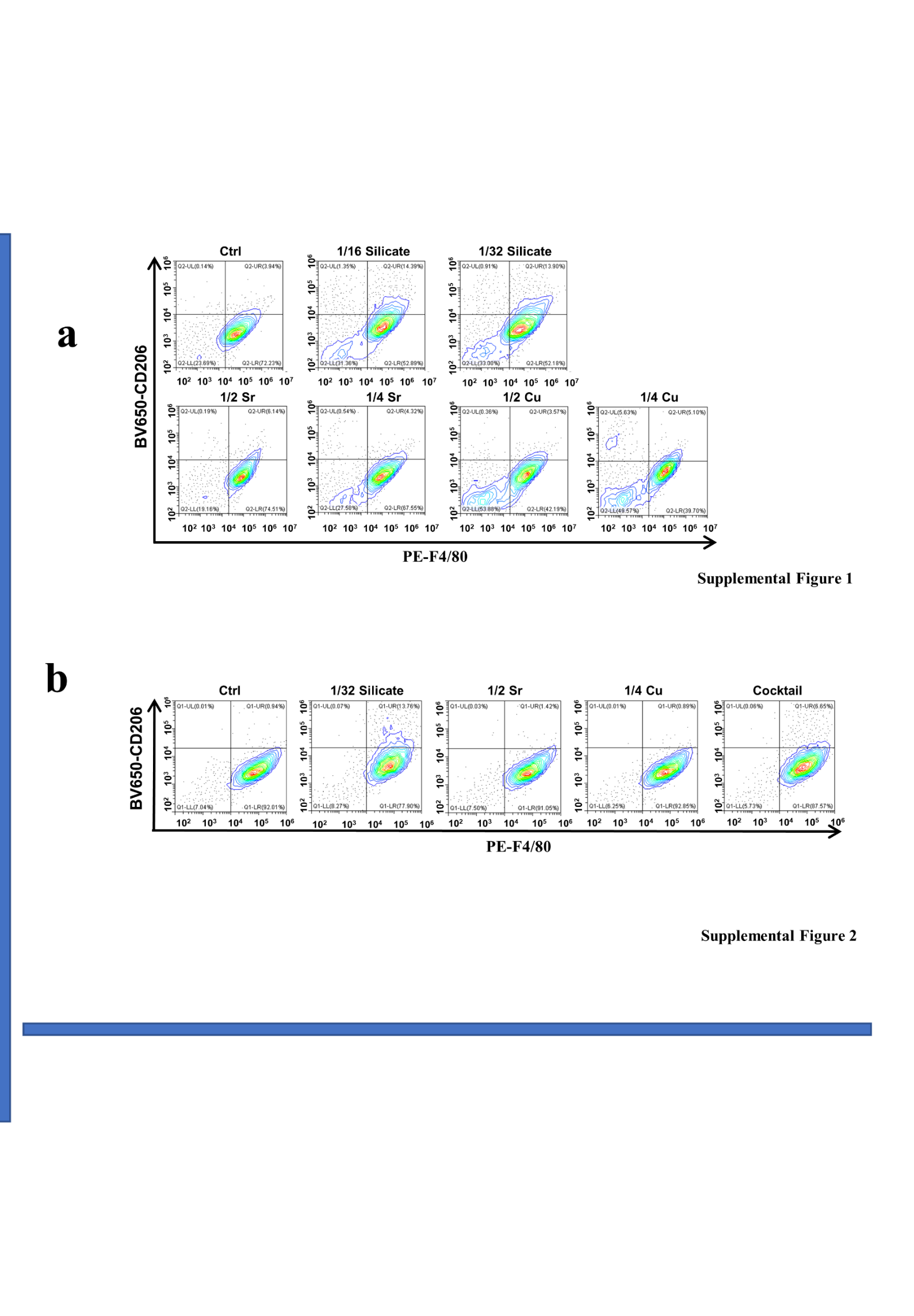


**Figure S11** Representative flow cytometric images of M2-type polarization of MBMM (presented as the percentage of CD206^+^F4/80^+^ cells) treated with single ions and the ion cocktail for 48 h using phenotypic markers F4/80 (M0) and CD206 (M2). Ctrl, control medium.


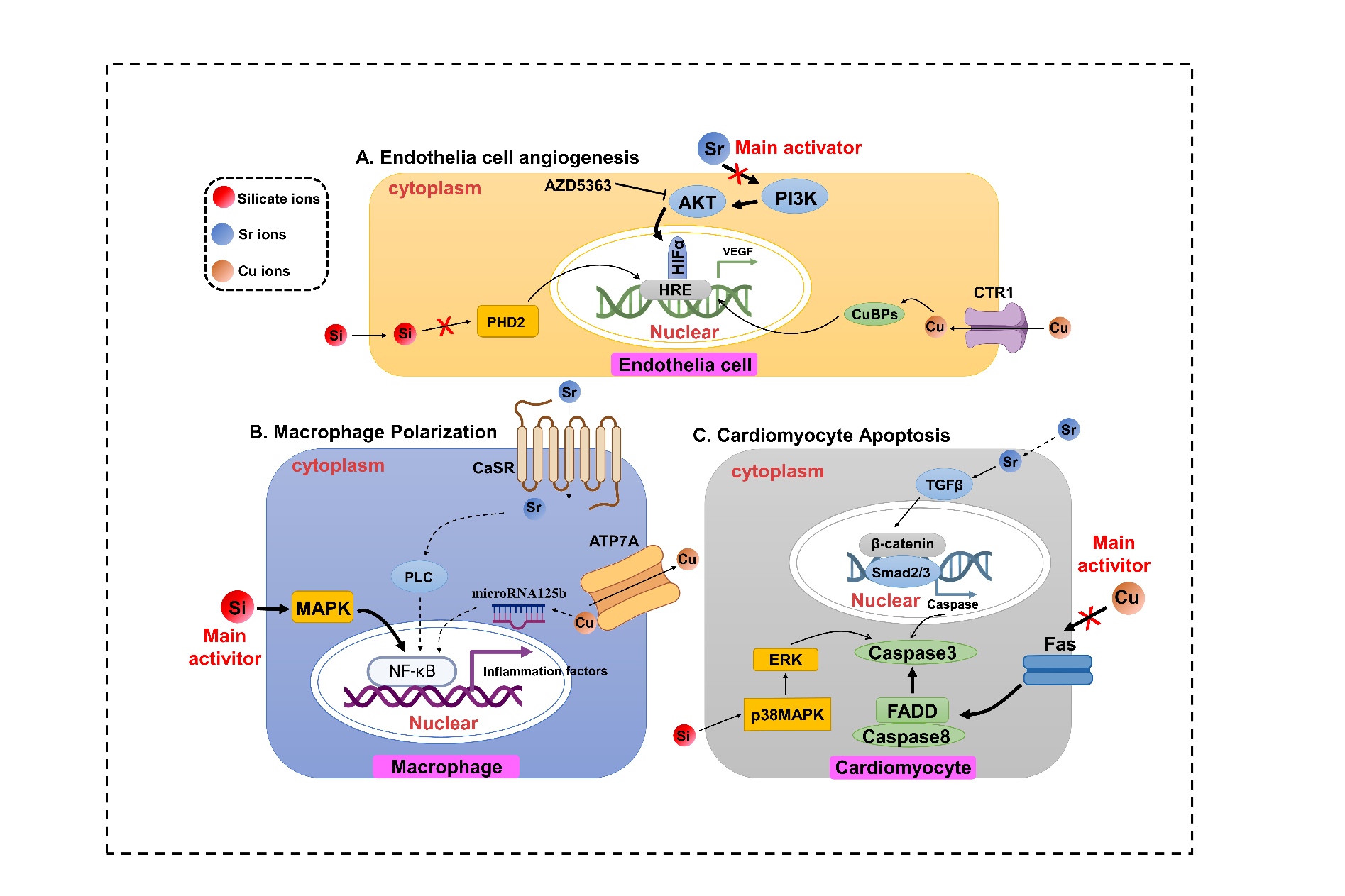


**Figure S12** The possible mechanism of ion cocktail in synergistically regulating endothelia cell, macrophage, and cardiomyocyte in the treatment of MI. **A)** Single Sr ions has the strongest stimulatory effect on promoting angiogenesis among three ions by activating AKT-HIF1α-VEGF signaling pathway, while Cu and silicate ions attribute to the synergetic effect of the ion cocktail by stimulating CTR1-CuBPs-HIF1α-VEGF and PHD2-HIF1α-VEGF pathway, respectively. **B)** Single silicate ions have the strongest stimulatory effect on inhibiting inflammation by regulating MAPK-NF-κB pathway, while Sr and Cu ions may attribute to the synergetic effect of the ion cocktail by regulating CaSR-PLC-NF-κB and ATP7A-microRNA125b-NF-κB pathway, respectively. **C)** Single Cu ions has the strongest stimulatory effect on protecting myocardium by regulating FAS-FADD-caspase pathway, while Sr and silicate ions attribute to the synergetic effect of ion cocktail by stimulating TGFβ-smad2/3-caspase and p38MAPK-ERK-caspase pathway, respectively. Fig. S12 was created with BioRender.com.


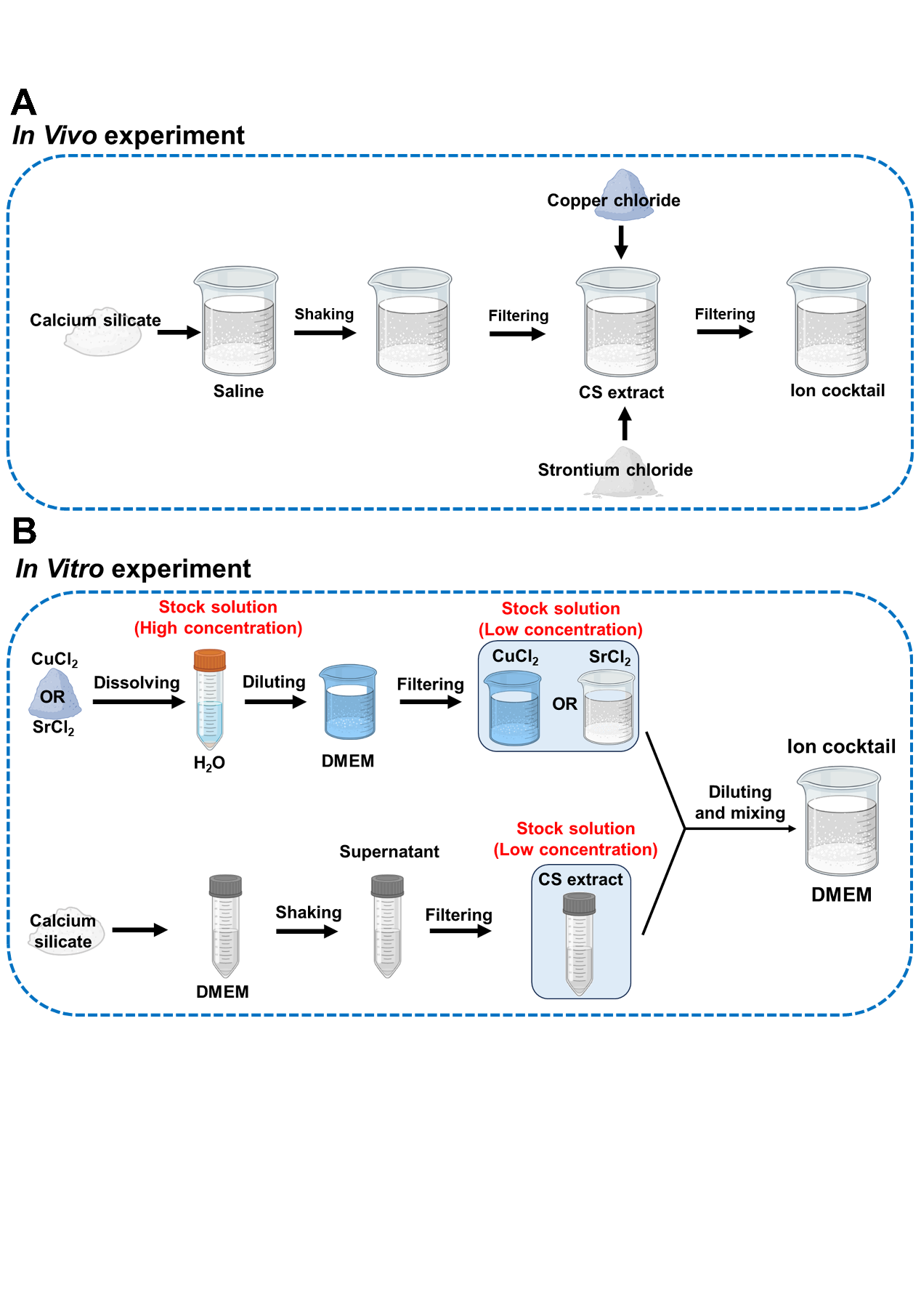


**Figure S13 A)** The flow chart of ion cocktail solution configuration for *in vivo* rat and Bama minipigs experiments. **B)** The flow chart of ion solution configuration for *in vitro* cell experiments. Fig. S13 was created with BioRender.com.

**Table S1** The value of APD90 and CaD90 for different areas of the heart (normal area, peri-infarct area or infarct area) in Sham, MI + Saline and MI + Cocktail groups. The values in red represent the average of each group.

**Table S2** The difference value (DV) of APD_90_ and CaD_90_ between different areas of the heart (infarct and normal area or infarct and peri-infarct area) in Sham, MI + Saline and MI + Cocktail groups. The values in red represent the average of each group.


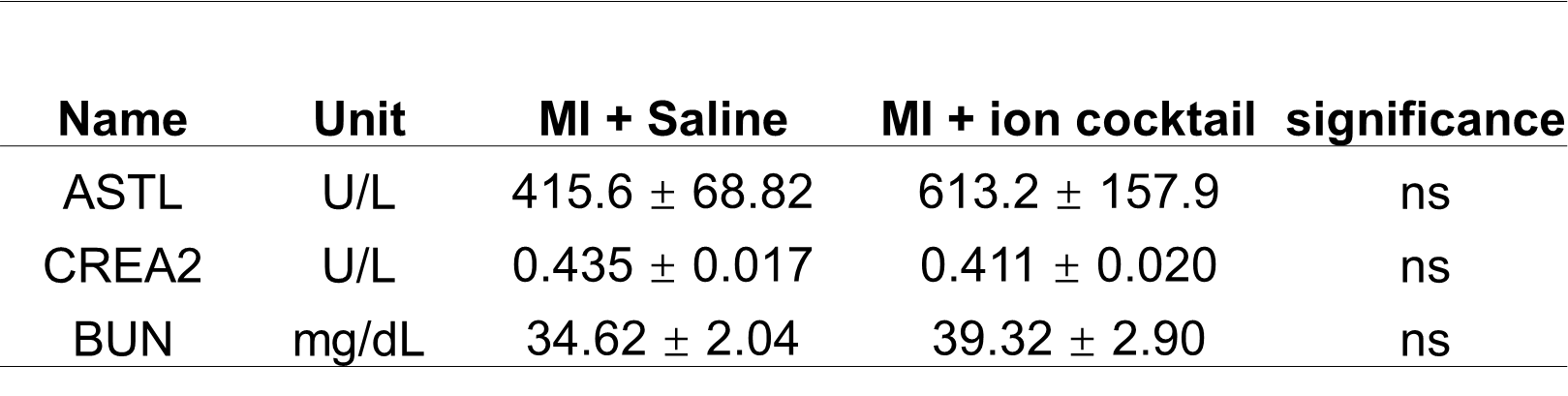


**Table S3** The value of aspartate aminotransferase (ASTL), creatinine (CREA), blood urea nitrogen (BUN) in rat serum between MI + Saline and MI + Cocktail groups. ns, no significance.

**Table S4** The ion concentration of the Si, Sr and Cu at different dilution ratio.


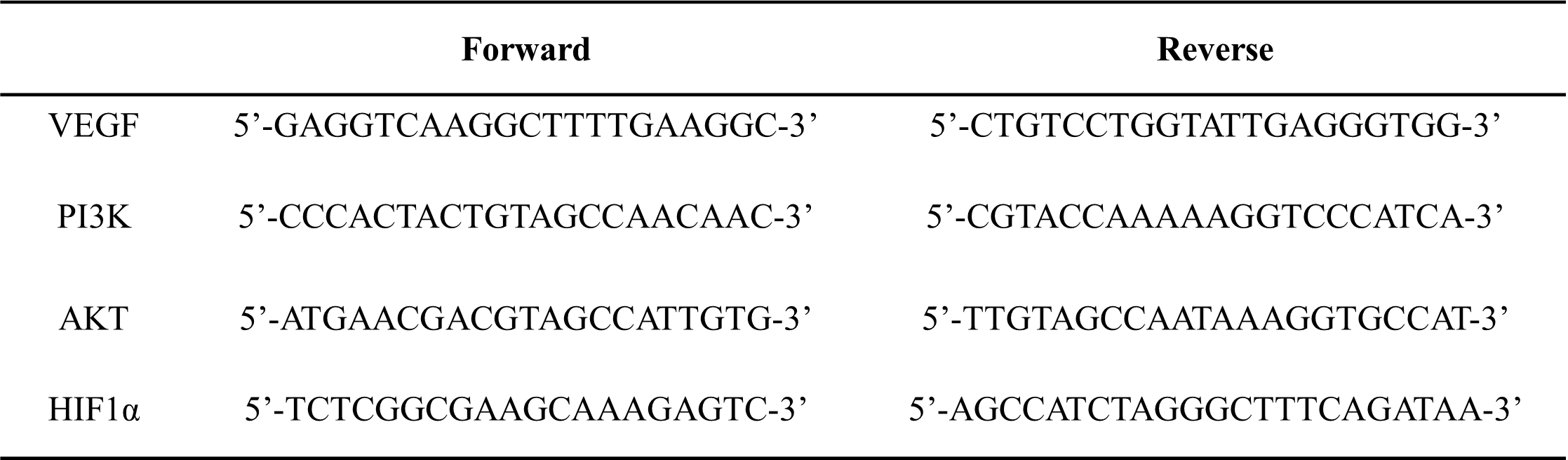


**Table S5** The primer sequence used in this study.
